# Analyzing the impact of subcutaneous injection needle, device, and administration characteristics on patient pain, anxiety, and safety: a systematic literature review

**DOI:** 10.1080/10717544.2026.2719057

**Published:** 2026-08-27

**Authors:** Mehul Desai, Keith Candiotti, Aaron Pinkhasov, R. Brandon Hunter, Harman Chopra, Lisa Gorski, Samantha Shenoy

**Affiliations:** a Enable Injections, Inc., Cincinnati, OH; b University of Miami, Miller School of Medicine, Miami, FL; c New York University Grossman Long Island School of Medicine, Mineola, NY; d Texas Children's Hospital, Houston, TX; e Weill Cornell Medicine, Anesthesiology, Pain, and Critical Care Medicine, New York, NY; f Compassus, Brentwood, TN; g Hematology, Blood & Marrow Transplant and Cellular Therapy (HBC) Program, University of California, San Francisco, CA

**Keywords:** Subcutaneous, injection, needle, needle gauge, needle bevel, needle length, pain, fear, anxiety, safety

## Abstract

The subcutaneous (SC) injection route is a commonly used and important method for therapeutic delivery of a wide range of compounds, and needle characteristics have a significant influence on patient pain, anxiety, safety, and other outcomes. This systematic review evaluates the evidence on how needle-specific characteristics (e.g. gauge, length, tip design, wall thickness, concealment) and administration- or device-related factors can affect patient-reported outcomes and clinical safety indicators during and following SC injections. A comprehensive search was conducted in MEDLINE, PubMed, Embase, and ClinicalTrials.gov in June 2024. Studies were included if they assessed the relationship between needle characteristics and pain, anxiety, safety, or related outcomes in individuals receiving SC injections. A dual-reviewer process was used for study selection, data extraction, and quality assessment. Sixty-two studies met inclusion criteria. Evidence consistently indicated that thinner and shorter needles reduced patient-reported pain and adverse events such as bruising and bleeding. Tapered and lubricated needles, hidden or retractable needle designs, and use of autoinjectors or prefilled syringes also contributed to reduced anxiety and improved user satisfaction. However, results were heterogeneous, and many studies lacked sufficient power or single-variable evaluation of individual needle parameters, limiting definitive conclusions. Needle characteristics significantly influence patient experience and safety with SC delivery. While both clinical evidence and practical experience clearly favor thinner, shorter, and concealed needles, further standardized, high-quality research is needed to isolate and quantify the specific contributions of individual needle characteristics to optimize injection practices and support patient-centered device design.

## Plain language summary

Fear of needles and pain from injections are common, and they can lead people to put off or avoid the treatment they need. Many medicines are given as an injection just under the skin, and the type of needle and device used can shape how much the injection hurts, how anxious it makes people feel, and how safe it is. We reviewed the published evidence on this question, bringing together findings from many studies.

Across these studies, thinner and shorter needles were generally linked to less pain and fewer problems such as bruising and bleeding. Needles with tapered tips also tended to be more comfortable. Devices that keep the needle out of sight, such as pen injectors, autoinjectors, and wearable injectors, helped reduce fear and were often preferred, and people who feel anxious about injections especially valued a hidden or retractable needle.

One challenge is that the studies were quite different from one another, and many tested several needle or device features at the same time. This makes it hard to say exactly how much each individual feature matters on its own. Pain was studied most often, but a good amount of research also covered safety, such as bruising and bleeding, along with a number of studies on anxiety.

Even so, the overall direction was consistent. Designing needles to be thinner, shorter, more comfortable, and less visible makes injections easier to tolerate, which may help patients stay on treatments they take for years. More carefully designed studies are needed to confirm how much each feature contributes.

## 1. Introduction

Biologics such as antibodies treat a range of conditions. While traditionally administered intravenously (IV), subcutaneous (SC) administration is an increasingly accepted route of administration and offers comparable or improved safety and efficacy. SC administration reduces treatment burden, improves quality of life and convenience, lowers healthcare costs and resource use, increases patient throughput, and benefits patients with vascular exhaustion or poor venous access. (Launay-Vacher 2013; Pivot et al. 2014; De Cock et al. 2016; Bittner et al. 2018; Epstein 2021) Several SC biologics are approved and in clinical development across therapeutic areas, including endocrinologic and metabolic diseases, immunology, dermatology, and oncology (Mathias et al. 2024).

The rapid growth of SC formulations has accelerated advancements in both needle engineering and broader drug delivery systems to reduce leakage, tissue damage, back-flow, and excessive tissue pressure, (Schneider et al. 2023) largely driven by the need to improve patient and healthcare practitioner (HCP) experience and treatment adherence and reduce costs and healthcare resource utilization. (Walsh et al. 2007; Pivot et al. 2013; Martin et al. 2013; Franken et al. 2020; Heinemann 2023) Needle-specific design features include gauge (diameter), length, wall thickness, bevel geometry, tapering, and lubricity, while broader device- or administration-related characteristics include autoinjectors, prefilled syringes (PFSs), on-body delivery systems (OBIs, also called on-body delivery systems or wearable infusers), hidden or retractable systems, injection speed, pressure-sensitive delivery, and injection technique. Table 1 summarizes some of the impacts needle and device characteristics and SC administration factors may have across the healthcare continuum.

**Table 1.** Needle characteristics: impact across the healthcare continuum.

Domain	Needle, Administration, and Device-Related Factors	Possible Benefits	Possible Stakeholder Impacts
Patient Experience: Pain and Anxiety Reduction	Larger gauge (thinner needle) Shorter length (e.g. 4-6 mm) Lubricated tip and multibevel design Shielded/invisible needle designs Consistent needle sharpness	Reduced insertion pain Lower anxiety, especially in needle-phobic or treatment-naïve patients Reduced tissue trauma Increased comfort in frequent injections	Potential for improved adherence Reduced fear of injections Potential for better initiation in long-term therapies for chronic conditions (e.g. diabetes, autoimmune diseases)
HCP Usability and Safety	Safety needles (e.g., retractable, shielded) Fixed needle systems Flexible or precision-ground bevels Ergonomic grip/handling	Fewer needlestick injuries Easier, faster injections Reduced administration errors Less variability across users	Enhanced nurse safety More consistent technique across staff Lower HCP stress and training burden
Pharmacy and Supply Chain	Device compatibility (e.g., pens, PFS) Ready-to-use needle packaging Unit-dose formats with safety features	Reduced preparation time Streamlined patient counseling Fewer compatibility errors	More efficient dispensing Improved inventory management Safer patient handoff
Needlestick Injury Prevention	Passive safety mechanisms Auto-retracting/auto-shielding designs No need to recap manually Single-use engineering controls	Reduction in accidental needlesticks Training-independent protection Safer sharps disposal	Fewer occupational injuries Potential for reduced system costs (testing, follow-up) Opportunity for safer home and community care
Healthcare System-Level Outcomes	Combined innovations in design, safety, and comfort	Reduced complications and hesitancy Optimised therapy initiation and adherence More efficient workflows	Better clinical outcomes Lower long-term healthcare costs Improved satisfaction across patients, HCPs, and pharmacists

Abbreviations: HCP, healthcare provider; PFS, prefilled syringe.

Early reusable needles required heat sterilization and manual sharpening (Parker 1924; Heinemann 2023) and were long (19-26 mm), increasing the likelihood of inadvertent intramuscular injection, especially in children and self-injecting adults. (Gibney et al. 2010; Heinemann 2023) Variability in SC adipose thickness by sex, age, and body mass index (BMI) further contributed to uncertainty regarding optimal injection depth. (Lo Presti et al. 2012) Mean abdominal skin thickness in adults ranges from 1.3 to 3.1 mm (Akkus et al. 2012; Jain et al. 2013; Sim et al. 2014, 2014; Derraik et al. 2015; Van Mulder et al. 2017; Torii-Goto et al. 2023) and in children and adolescents ranges from 1.6 to 2.6 mm, (Lo Presti et al. 2012; Marran and Segal 2014; Derraik et al. 2015; Saitoh et al. 2015; Lim et al. 2018; Kodikara et al. 2020; Kaba et al. 2021) with thicker skin generally reported in boys and men (Gibney et al. 2010; Jain et al. 2013; Wang et al. 2016; Van Mulder et al. 2017) and in individuals with higher BMI. (Gibney et al. 2010; Wang et al. 2016) Improved understanding of demographic effects on skin layers (Shin and Kim 2006; Lo Presti et al. 2012, 2014; Hirsch et al. 2014; Derraik et al. 2015; Kodikara et al. 2020) led to development of shorter, thinner needles. (Gibney et al. 2010; Hansen and Matytsina 2011; O'Neal et al. 2015) Subsequent needle-specific features such as thin walls and advanced bevels were designed to further reduce penetration force. (Hirsch et al. 2012) Figure 1 shows illustrations of key needle characteristics, including bevels, tapers, and the relationship between wall thickness and interior diameter. Although thinner needles improve patient experience, concerns include leakage, injection force due to smaller internal diameter, and bending and breakage. (Præstmark et al. 2016a; 2016b) However, many of these concerns exist in systems that require manual administration.

**Figure 1.**
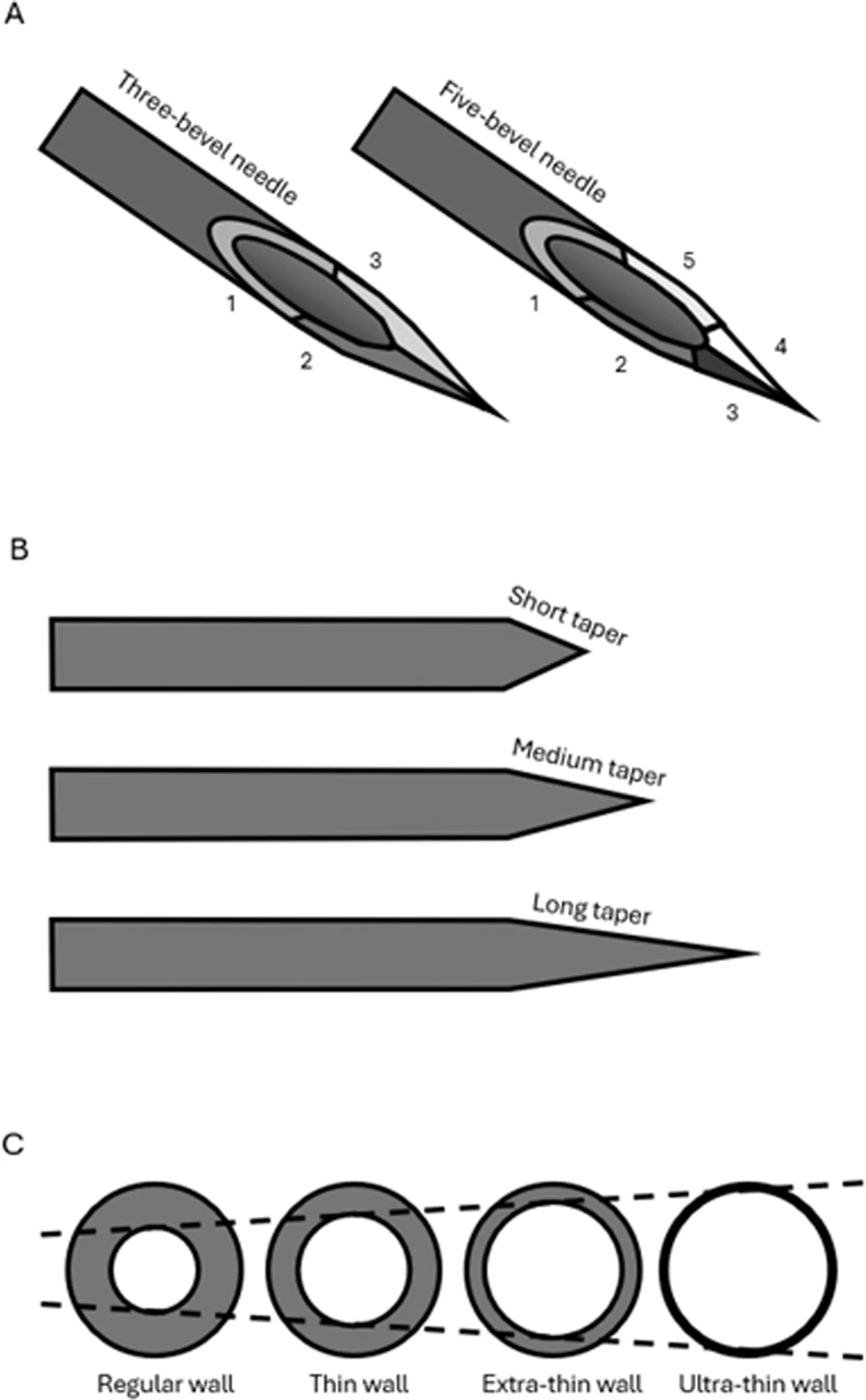
Needle Characteristics: A) Tip Bevels, B) Tapering, and C) Relationship between wall thickness and interior diameter.

**Figure 2.**
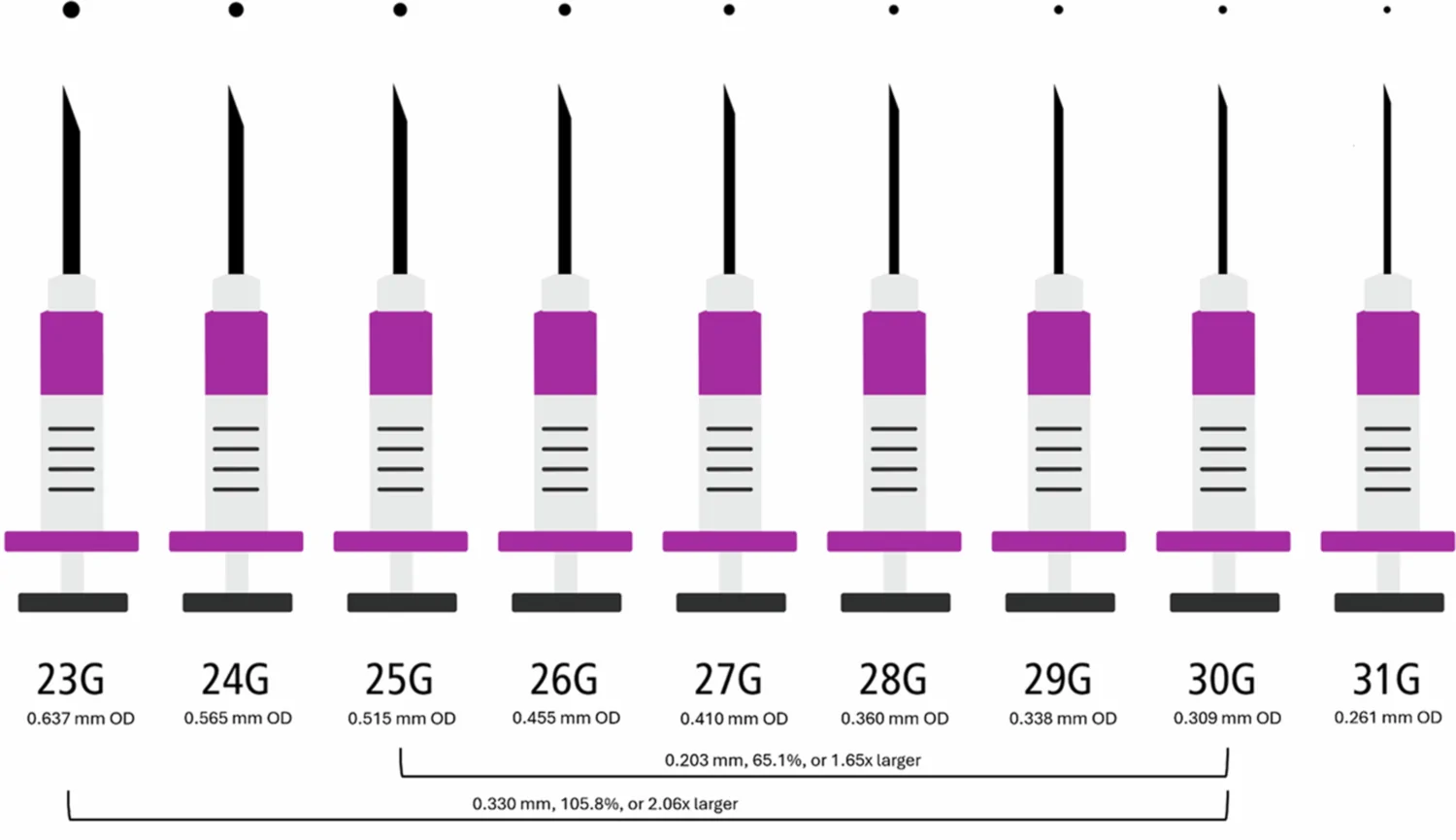
Comparison of needle gauges relevant to common clinical experiences. Abbreviations: G, gauge; mm, millimeters; OD, outer diameter. Needle gauge sizes ranging from 23 G to 31 G are shown with their corresponding outer diameters. (International Organisation for Standardisation (ISO) 2016) Syringes are not drawn to scale due to space restrictions. To aid visualization, a 23 G needle is similar in size to many household sewing pins, while a 31 G needle is similar in diameter to a typical filiform acupuncture needle; however, because the dimensions of everyday objects vary, these comparisons are illustrative only.

**Figure 3.**
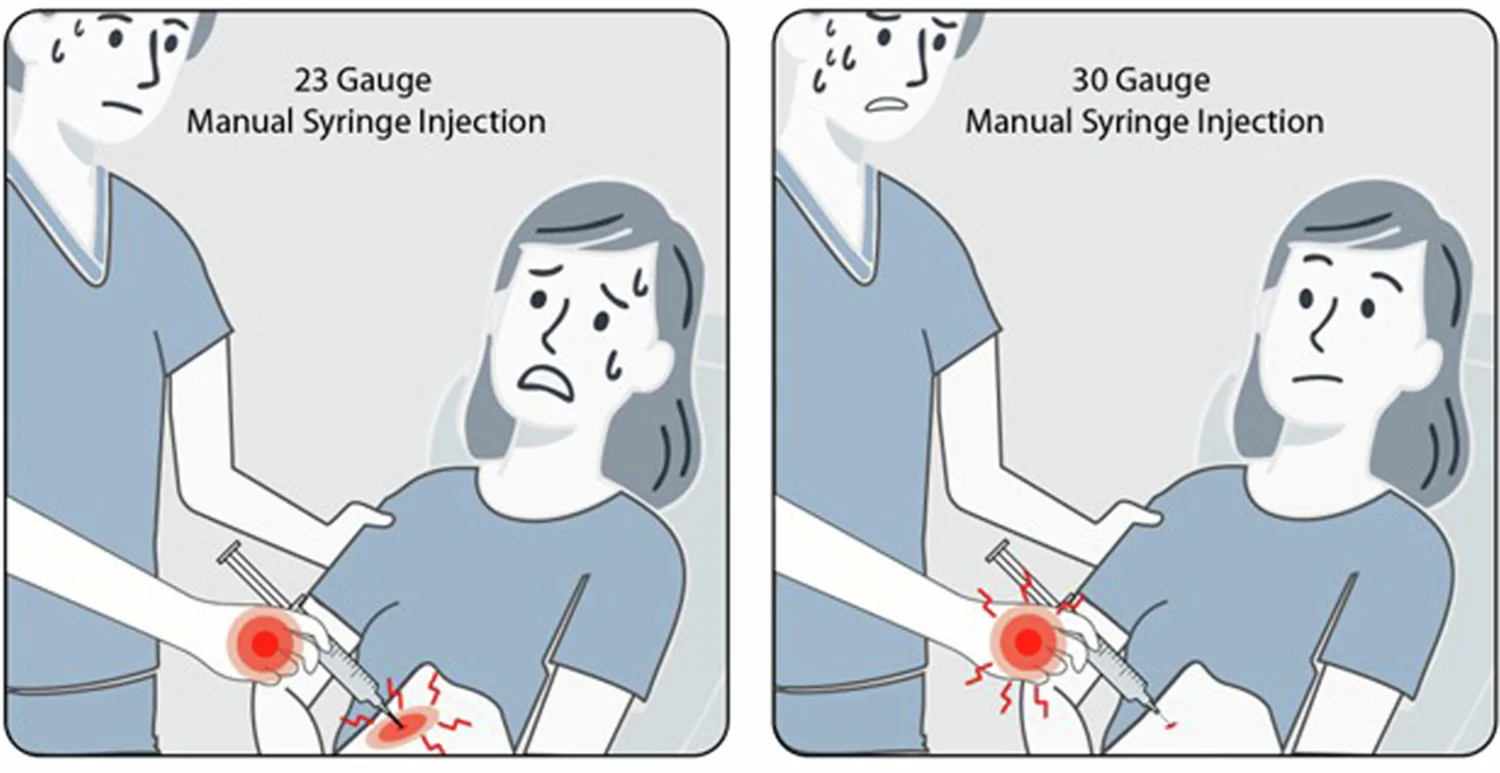
Patient and healthcare provider (HCP) experience comparison: 23-gauge vs. 30-gauge needle for manual subcutaneous injection. This illustrated comparison shows the difference in patient discomfort between 23-gauge (left panel) and 30-gauge (right panel) needles during manual syringe injection for HCP-assisted drug delivery. A skin pinch-up method is typically used for subcutaneous injections but is not shown in this illustration to preserve the depiction of pain at the injection site. The 23-gauge injection causes significant patient pain, tissue trauma, and distress, but requires less force for the HCP to depress the plunger. In contrast, the 30-gauge injection provides markedly improved patient comfort and tolerability, but the substantially smaller needle diameter creates higher backpressure during injection, requiring considerably more manual force from the HCP and increasing physical strain and repetitive injury risk.

Importantly, changes between needle gauges are nonlinear; small increases in gauge (i.e. thinner needles) can meaningfully affect injection force and pain (Figure 2). For example, 23–25 G (0.642–0.515 mm) (Hamilton 2026) needles, which are used for some SC injections, (Wolicki and Miller 2026) are 21.6% thinner than 21 G (0.819 mm) (Hamilton 2026) venipuncture needles, but are 36–59% thicker than the 27/30 G (0.413/0.312) (Hamilton 2026) needles commonly used for facial botulinum toxin injections (Kämmerer et al. 2024) and 44–67% thicker than the 28/30/33 G (0.362/0.312/0.210 mm) (Hamilton 2026) lancets commonly used for blood sugar glucose monitoring. (Mianowska et al. 2021) Given that blood draws are a recognized source of procedure-related pain and anxiety, (McLenon and Rogers 2019) the use of needles only modestly smaller than those used for venipuncture may contribute to patient discomfort with some SC therapies. These comparisons illustrate how seemingly small differences in gauge number correspond to substantial differences in physical diameter and may therefore influence both patient and HCP experience (Figure 3). The challenges shown in Figure 3 occur during the delivery of large-volume subctuaenous biologics; fortunately, they can be alleviated by delivery devices that utilize hands-free administration such as infusion pumps and on-body injectors.

Figure 3 needle fear, anxiety, and phobia are common, with one systematic review of 119 studies (meta-analysis of 35) finding needle fear in most children, 20–50% of adolescents, and 20–30% of adults aged 20–40 years. (McLenon and Rogers 2019) A global survey of 2,098 adults reported needle phobia in 63.2%, leading to avoidance of blood draws (52.2%), donation (49.0%), and vaccination (33.1%). (Alsbrooks and Hoerauf 2022) One study of hematology/oncology nurses found that 88.89% reported additional burden due to counseling needle-phobic patients, 50.00% reported delayed appointments, and 33.30% reported missed appointments. (Desai et al. 2025) Smaller or hidden needles, distraction or relaxation techniques, education, and therapy have all been reported to reduce fear (Alsbrooks and Hoerauf 2022) and may therefore improve patient adherence.

Injection pain is a leading contributor to needle fear. (Alsbrooks and Hoerauf 2022) Injection site pain can contribute to distress, poor adherence, and worsening anxiety or phobia. (Berlin et al. 1997; Alsbrooks and Hoerauf 2022) Multiple factors impact pain following SC injection, including both needle-specific characteristics (e.g. needle length, diameter, bevel geometry, lubricity, and visibility) and broader administration-related variables such as injection site, angle, injection speed or flow rate, and biologic characteristics such as temperature, volume, viscosity, pH, and excipients. (Usach et al. 2019; St Clair-Jones et al. 2020; Junker et al. 2022) Injection pain has also been shown to decrease patient adherence. (Gely et al. 2020)

SC injections may also produce adverse events such as erythema, pruritus, edema, bruising and hematoma, induration, hypersensitivity reactions, cellulitis-like reactions, and infections that contribute to pain and anxiety. (Kim et al. 2023) However, SC administration is well-suited for the management of chronic diseases, including cancers and autoimmune diseases. (Bittner et al. 2018) Transitioning from IV to SC can offer well-established advantages: decreased reliance on HCP administration, reduced healthcare costs and treatment burdens, fewer infusion-related reactions, more consistent plasma biologic concentrations, and improved patient experience and tolerability, with comparable efficacy and pharmacokinetics. (Kobrynski 2012; Pivot et al. 2014; Stoner et al. 2014; Merz et al. 2015; Fathallah and Balu-Iyer 2015; Rummel et al. 2017; Mateos et al. 2019; Chari et al. 2019; Johnson et al. 2019; Stewart et al. 2020; Badkar et al. 2021) Slowing IV infusion to mitigate sensitivity reactions aligns with the pharmacokinetic effects of SC dosing, and it has therefore been proposed that when peak concentration (C_max_)-related reactions occur with IV infusion, shifting to SC may be advantageous. (Mao et al. 2013; Bittner et al. 2018) SC immunoglobulin’s steady-state kinetics, with lower systemic absorption and absence of post-infusion spikes, are also associated with fewer severe systemic reactions than IV immunoglobulin. (Ballow 2007; Kobrynski 2012) In a Phase 3 trial of isatuximab SC vs. IV in multiple myeloma, infusion reactions were reported in 1.5% SC using an OBI vs. 25.0% IV (~17-fold difference). (Ailawadhi et al. 2025) Similarly, in the COLUMBA trial of daratumumab/hyaluronidase, injection site reactions occurred in 7% SC using a manual push syringe, while infusion-related reactions occurred in 35% IV (5-fold difference). (Mateos et al. 2020) Although these findings support the broader advantages of SC delivery systems, they should not be interpreted as attributable to needle characteristics alone.

**Figure 4.**
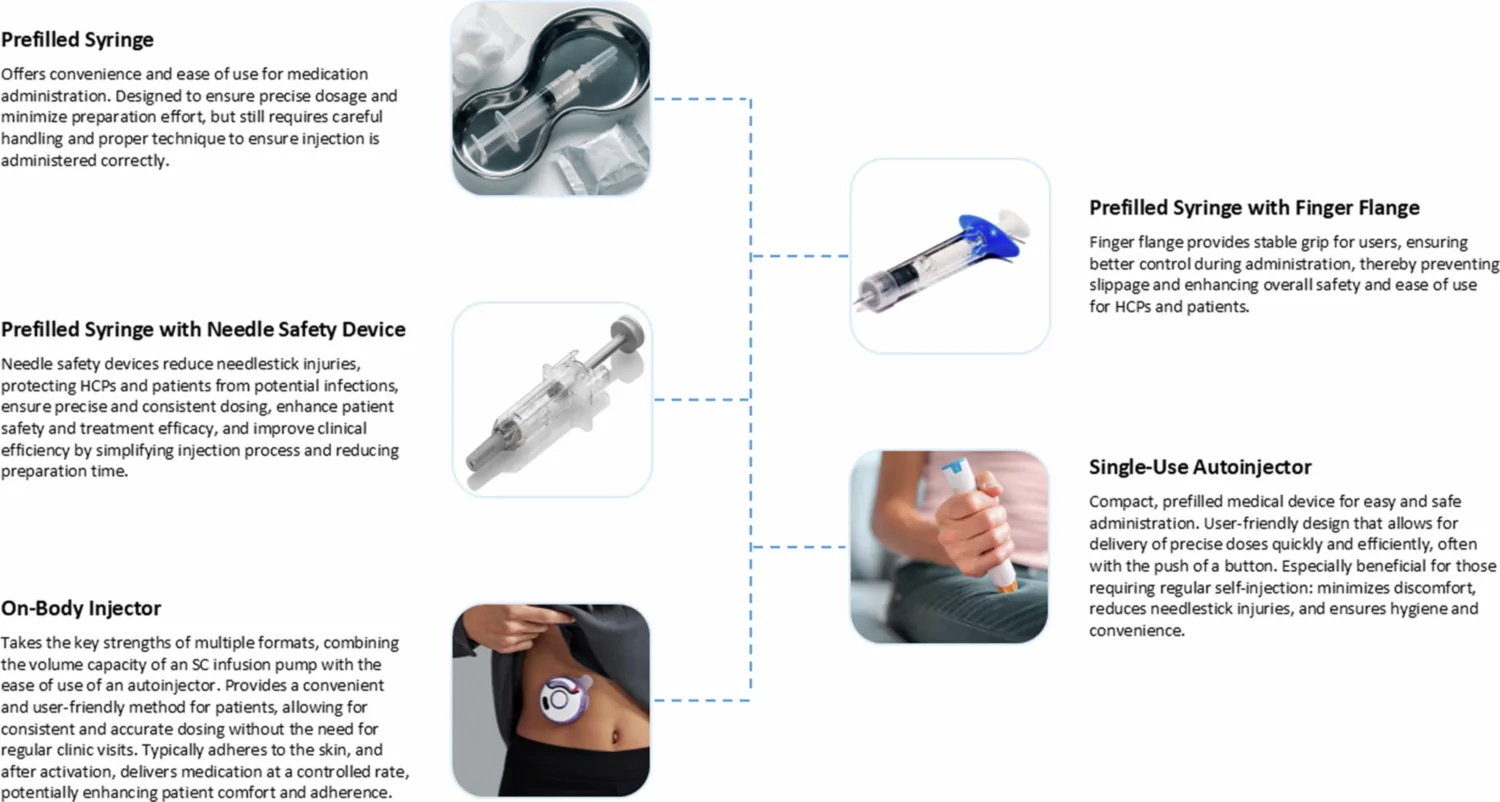
Currently available subcutaneous devices. Abbreviations: HCP, healthcare provider.

SC biologics can be administered with various devices (Figure 4), including PFSs, single-use autoinjectors, and OBIs. These systems differ not only in needle design but also in delivery mechanics, injection speed, automation, user handling, and needle visibility. PFSs require substantial manual effort dependent on the biologic viscosity, offer less consistent administration, and have exposed needles. They may come with finger flanges, which require manual injections and also have exposed needles, and/or with needle safety devices, which offer easier manual injections and hidden needles. Single-use autoinjectors are limited in biologic administration volume, must be held at the site of administration, require a custom biologic container, use hidden 25–29-gauge needles, and may be associated with more pain. Finally, OBIs are hands-free, sometimes use the original biologic vial, and employ a small, hidden, retractable, 29–30-gauge needle that may be less painful. Newer SC devices can reduce needle visibility and phobia compared with visible-needle SC systems and IV infusions. (Stevenson et al. 2024) Autoinjectors and syringes increasingly include integrated safety mechanisms such as auto-retraction, needle shields or guards, or lockouts valued by patients for reducing administration errors and accidental injuries and for HCPs for lowering occupational sharps exposure. (Stevenson et al. 2024)

In summary, needle-related pain, anxiety, and safety influence adherence and clinical outcomes. (Zambanini et al. 1999; Peyrot et al. 2005; Turner et al. 2009) However, outcomes associated with SC administration are likely influenced by a combination of needle-specific physical characteristics and broader device- and administration-related factors. Prior reviews have not focused exclusively on SC injections (McLenon and Rogers 2019) or have emphasized pain while overlooking anxiety or safety. (Usach et al. 2019) The objective of this systematic review was therefore to synthesize evidence on how SC needle characteristics and related SC delivery factors influence pain, anxiety, and safety to optimize injection practices and patient-centric SC device development.

## 2. Methods

### 2.1. Eligibility criteria

This systematic review was conducted and reported according to PRISMA 2020 guidance. Studies eligible for inclusion in this systematic review were those evaluating the impact of needle-specific characteristics (e.g. diameter, length, bevel tip) or administration- (e.g. needle angle, insertion velocity) or device-related factors (e.g. device choice, shield material, hidden or concealed mechanism) on pain, anxiety, and safety in individuals receiving SC injections.

The review was originally designed to evaluate the relationship between needle characteristics and patient outcomes associated with SC administration. However, during screening and data extraction it became evident that many studies did not evaluate needle characteristics in isolation. Instead, needle properties were commonly embedded within broader injection systems, including hidden-needle designs, safety mechanisms, autoinjectors, prefilled pens, and administration-related features. Because these factors may independently or jointly influence outcomes such as pain, anxiety, fear of injection, usability, and treatment preference, restricting inclusion to studies evaluating needle characteristics alone would have limited the applicability of the findings and excluded a substantial body of relevant evidence. The scope of the review was therefore expanded to capture device- and administration-related characteristics that contribute to the overall patient injection experience. Where possible, results were synthesized according to the specific intervention evaluated; however, the review also highlights instances in which the effects of needle characteristics could not be disentangled from broader device or administration features.

Inclusion criteria were as follows: patients or healthy volunteers; in-clinic/hospital or at-home administration; SC injections administered by HCPs or patients/participants; published articles evaluating the impact of needle characteristics on pain, anxiety, and/or safety within the context of SC injections of active or inactive solutions (i.e. biotherapeutics or placebo solutions); articles published in English; and published articles/studies (e.g. randomized controlled trials, observational studies, case studies). Exclusion criteria were as follows: preclinical or animal studies; published articles detailing the impact of SC injection variables outside of the needle and/or outcomes other than pain, anxiety, and/or safety; intramuscular, intradermal, IV, or needle-free administration of active or inactive solutions (e.g. biotherapeutics or placebo solutions); articles published in a language other than English; and grey literature. Articles without full-texts available were also excluded. Review articles identified during screening were retained for background context and reference-list screening only and were not included in the systematic review study selection, data extraction, risk-of-bias assessment, or evidence synthesis. Accordingly, no review articles were included in the analyses presented in this manuscript.

### 2.2. Information sources

Information sources used to complete this systematic review included MEDLINE, PubMed, Embase, and ClinicalTrials.gov. Databases were searched from inception to the date of searches.

### 2.3. Search strategy

Searches were conducted on 20 June 2024. Search strategies are available in **Supplementary Table 1**.

### 2.4. Selection process

Databases were searched from inception to the date of the search, and search alerts were put in place to collect any additional records published during the systematic review and manuscript preparation process. Two individuals independently screened all studies retrieved in searches, removed duplicates, and excluded studies based first on title, then abstract, then full-text. Reference lists from selected articles were also screened. Any differences in the screening results were discussed in order to reach a consensus regarding full-text article inclusion in the review. After both individuals determined the full-text articles eligible for inclusion, both individuals used a semi-structured data collection form to participate in manual data extraction. Where necessary and feasible, attempts were made to contact the authors of included studies for any missing information or clarification required.

### 2.5. Data collection process

The following data were collected in an Excel file: title and year of publication, author(s), journal, inclusion and exclusion criteria, study methods and setting, population and participant characteristics, intervention(s), outcome(s), results, and number of and reasons for withdrawals and/or dropouts.

### 2.6. Risk of bias assessment

Risk of bias in individual studies was evaluated with the Revised Cochrane risk-of-bias tool for randomised trials (RoB 2), (Sterne et al. 2019) the Risk Of Bias In Non-randomised Studies - of Interventions (ROBINS-I) (Sterne et al. 2016) tool for non-randomised interventional studies, the Newcastle-Ottawa Scale (NOS) (Wells et al. 2013) for cohort and case-control studies, the Joanna Briggs Institute (JBI) critical appraisal checklist for analytical cross-sectional studies, (Briggs Institute 2024) and the JBI Critical Appraisal Checklist for Case Series (Munn et al. 2020) for case series studies. The Risk-of-bias visualisation (robvis) tool (McGuinness and Higgins 2020) was used to generate summary visualizations of the ROBINS-I and RoB 2 assessments.

Although the protocol proposed assessment of certainty of evidence using the GRADE framework, formal GRADE evaluation was not undertaken. During the review process, the scope was expanded to include device- and administration-related factors in addition to needle characteristics, resulting in numerous heterogeneous intervention-outcome comparisons across diverse populations, study designs, comparators, and outcome measures. Application of GRADE would therefore have required a large number of separate evidence profiles, many informed by only a small number of studies. Instead, confidence in the evidence was assessed qualitatively by considering study design, aggregated risk-of-bias assessments, consistency of findings, directness of evidence, and methodological limitations.

## 3. Results

### 3.1. Study selection

**Figure 5.**
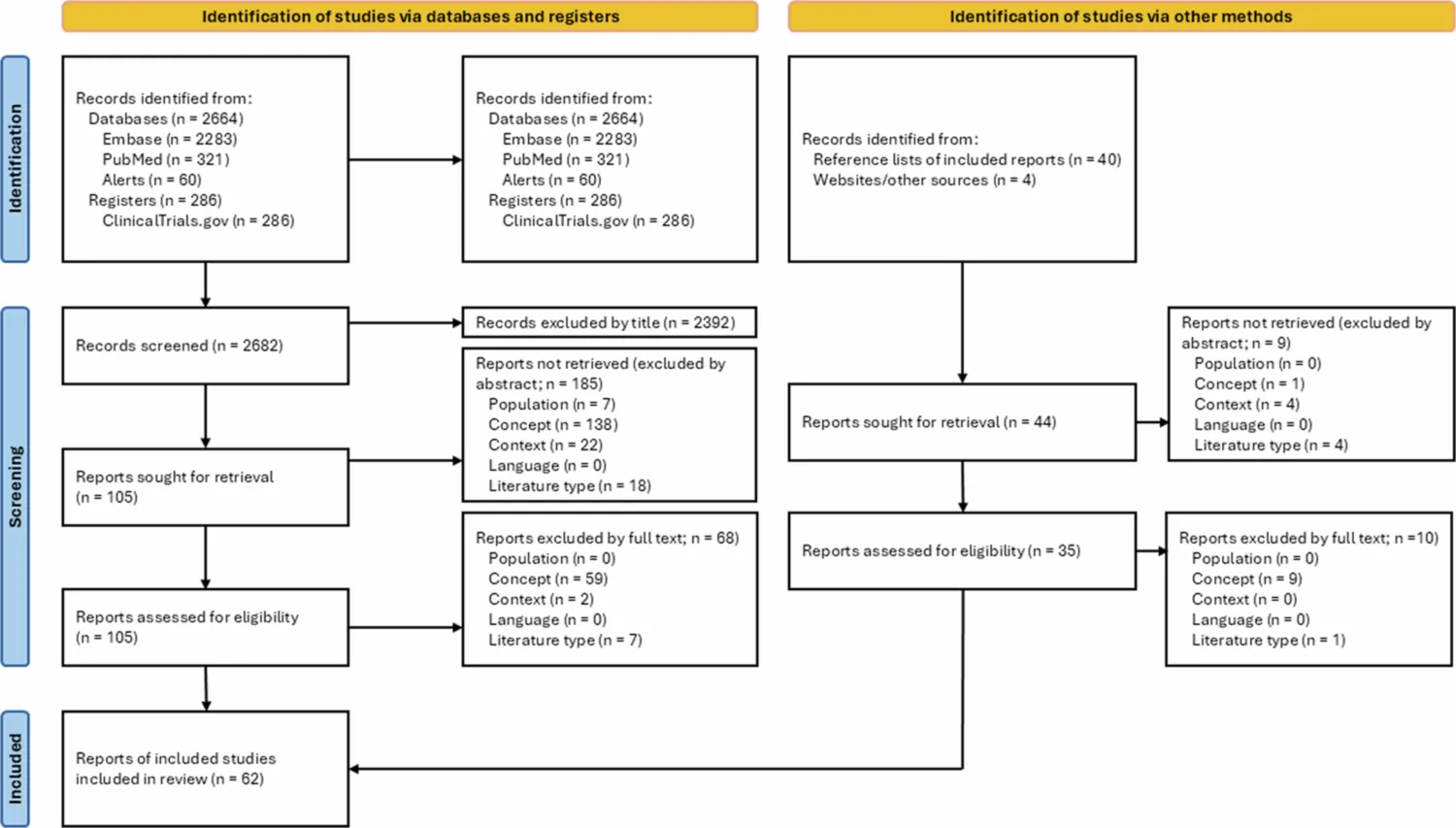
PRISMA flow diagram.

Initial searches resulted in 2726 hits after removing duplicates. After screening by title and abstract, 140 articles were selected. Of these, a further 79 articles were removed after reviewing the full-text articles. A total of 62 studies were reviewed and are included in this review. Figure 5 provides a PRISMA Flow Diagram summary of the selection process.

### 3.2. Study characteristics

Study designs varied widely and included randomized and unrandomized, open-label and blinded, single and parallel group, prospective and retrospective, observational studies and clinical trials. Participants included healthy volunteers, (Egekvist et al. 1999; Arendt-Nielsen et al. 2006; Gill et al. 2008; Jaber et al. 2008; Candiotti et al. 2009; Wågø et al. 2016; Zhang et al. 2021; Dang et al. 2024) patients in palliative care, (Youssef and Atkinson 1990; Currow and Cooney 1994; Macmillan et al. 1994; Abbas et al. 2005; Neo et al. 2016) and patients with diabetes, (Kadiri et al. 1998; Ross et al. 1999; Strauss et al. 1999; Hanas et al. 2000; Schwartz et al. 2004; Asakura et al. 2006; Kreugel et al. 2007; Miyakoshi et al. 2007; Iwanaga and Kamoi 2009; McKay et al. 2009; Siegmund et al. 2009; Hirsch et al. 2010, 2011; Hirsch et al. 2012; Chen et al. 2012, Ignaut and Fu 2012; Miwa et al. 2012; Aronson et al. 2013; Nagai et al. 2013; Bergenstal et al. 2015; Xia et al. 2015; Berard et al. 2015; Præstmark et al. 2016; Al Hayek and Al Dawish 2021; Al 2021) multiple sclerosis, (Jaber et al. 2008; Glenski 2009; D'Arcy et al. 2012; Masid et al. 2015; Devonshire et al. 2016) rheumatoid arthritis or other rheumatic disease, (Kivitz et al. 2006; Borrás-Blasco et al. 2010; Müller-Ladner et al. 2010; Striesow and Brandt 2012; Saraux et al. 2019; Alten et al. 2020; Lartey 2022) postoperative thromboembolism, (Coley et al. 1987) HIV, (Boyd et al. 2008) myocardial infarction, (Robb and Kanji 2002) growth disorders, (van Dommelen et al. 2024) anesthetic use, (Omoigui et al. 2006) chronic kidney disease, (Lim et al. 2012) cancer, (Dawkins et al. 2000) thalassemia, (Chan et al. 2004) musculoskeletal complaints, (Narvarte and Rosset-Llobet 2011) coronary artery bypass graft surgery, (Coley et al. 1987) multiple chronic diseases, (Pivato et al. 2024) or unspecified indication, (Lamblet et al. 2011) with two studies including groups of both healthy volunteers and patients with diabetes (Lee et al. 2018) or multiple sclerosis. (Jaber et al. 2008) Included studies recorded pain using ‘yes’ or ‘no’ criteria, on qualitative scales (e.g. ‘a lot better’ ‘a little better’ etc., versus the comparator) or used visual analogue scales (VAS) of varying types (e.g. 0–100 mm or 0–150 mm where 0 represents ‘no pain’ and 100 or 150 represents ‘worst pain imaginable’) or other numeric scales (10-unit numeric scale where 1 = no pain and 10 = highest pain). Due to the heterogeneity of the evidence available, meta-analysis was not possible and a narrative approach to evidence synthesis was selected.

### 3.3. Risk of bias in included studies

Results of the risk of bias assessment can be found in **Supplementary Tables 12** **–14** and **Supplementary Figures 1** **–4**. Across the 62 included studies, the overall methodological quality varied. Non-randomized studies (**Supplementary Figures 1 and 2**) had a serious risk of bias across most domains, primarily due to uncontroled confounding, non-random participant selection, and a lack of blinding, prespecified analysis plans, or adjustment for baseline differences. Randomized trials (**Supplementary Figures 3 and 4**) generally showed some concerns overall, with low risk of bias related to randomization or missing data but frequent lack of blinding and limited preregistration, resulting in potential performance and reporting biases in studies with subjective endpoints such as injection pain and usability. Several cross-sectional studies (**Supplementary Table 12**) were included and generally demonstrated moderate to serious risk of bias, reflecting the inherent limitations of descriptive, self-reported designs (i.e. confounding, selection bias, lack of temporality), although their outcome measurements were typically standardized. The single prospective cohort study (van Dommelen et al. 2024) (**Supplementary Table 13**) was judged as being of good quality, with a representative cohort and objective outcome assessment, but limited adjustment for confounders. The only case series study (Abbas et al. 2005) (**Supplementary Table 14**) had unclear to low risk of bias in measurement and analysis but lacked detailed clinical reporting, which limits external validity. Collectively, while the included randomized trials provided moderate-quality evidence for objective outcomes, the non-randomized and observational studies were limited by methodological weaknesses, and their results should therefore be interpreted with caution.

### 3.4. Results of Individual Studies

#### 3.4.1. Needle characteristics

##### 3.4.1.1. Needle length.

Across 24 studies evaluating needle length in 2,658 participants, most conducted in patients with diabetes, shorter needles were generally associated with improved patient experience, including reduced pain, greater comfort, reduced needle anxiety, stronger patient preference, and fewer injection-related adverse events (**Supplementary Table 2**). (Strauss et al. 1999; Robb and Kanji 2002; Schwartz et al. 2004; Chan et al. 2004; Kreugel et al. 2007; Gill et al. 2008; Iwanaga and Kamoi 2009; McKay et al. 2009; Hirsch et al. 2010, 2011; Narvarte and Rosset-Llobet 2011, 2012; Ignaut and Fu 2012; Miwa et al. 2012; Nagai et al. 2013; Bergenstal et al. 2015; Berard et al. 2015; Pager et al. 2020; Dang et al. 2024; van Dommelen et al. 2024; Al 2021) Outcomes assessed included pain, bleeding, bruising, leakage, fear, adherence, usability, and patient satisfaction.

Evidence was strongest in diabetes populations, where several studies reported lower pain scores and greater preference for shorter needles than for longer comparators. (Ross et al. 1999; Schwartz et al. 2004; Kreugel et al. 2007; McKay et al. 2009; Hirsch et al. 2010, 2012; Bergenstal et al. 2015; Al Hayek and Dawish, 2021) Shorter needles were also associated with reduced needle anxiety, (Bergenstal et al. 2015) reduced injection-related anxiety and improved ease of use, (Pager et al. 2020) and fewer injection-related adverse events, (Kreugel et al. 2007; Hirsch et al. 2012; Al Hayek and Dawish, 2021) including bleeding or bruising, in several studies. Strong patient preference for shorter needles was reported across multiple studies and age groups. (Strauss et al. 1999; Schwartz et al. 2004; McKay et al. 2009; Bergenstal et al. 2015).

However, findings were not uniform. Dang et al. found no clinically meaningful differences in pain or participant preference between 6-, 9-, and 12-mm needles in healthy volunteers, although the 12-mm needle was associated with more injection site reactions. (Dang et al. 2024) Similarly, Ignaut and Fu, Kreugel et al., Masid et al., and Robb and Kanji reported no significant differences in one or more assessed outcomes between needle lengths. (Robb and Kanji 2002; Kreugel et al. 2011; Ignaut and Fu 2012; Masid et al. 2015) Narvarte and Rosset-Llobet reported lower pain severity with a 12-mm mesotherapy needle despite pain occurring with a greater proportion of injections. (Narvarte and Rosset-Llobet 2011).

Interpretation of the evidence is complicated by substantial heterogeneity in study design and needle comparisons. Several studies compared needles that differed simultaneously in length, gauge, tapering, wall thickness, or other design characteristics, (Iwanaga and Kamoi 2009; Miwa et al. 2012; Nagai et al. 2013) limiting attribution of observed effects specifically to needle length. Furthermore, most evidence was derived from diabetes populations, with comparatively limited data available for other indications. Nevertheless, the overall direction of evidence generally favoured shorter, thinner needles for improving patient experience without compromising treatment delivery.

##### 3.4.1.2. Needle diameter.

Seventeen studies evaluated needle diameter in 3,810 participants (**Supplementary Table 3**). (Coley et al. 1987; Egekvist et al. 1999; Hanas et al. 2000; Robb and Kanji 2002; Chan et al. 2004; Arendt-Nielsen et al. 2006; Miyakoshi et al. 2007; Gill et al. 2008; Jaber et al. 2008; Boyd et al. 2008; Iwanaga and Kamoi 2009; McKay et al. 2009; Glenski 2009; Nagai et al. 2013; Præstmark et al. 2016; Wågø et al. 2016; van Dommelen et al. 2024) The evidence generally favoured smaller-diameter (higher-gauge) needles for reducing pain and improving patient-reported outcomes, although interpretation is complicated by frequent covariation with needle length, tapering, bevel design, wall thickness, and delivery system characteristics. Outcomes assessed included pain, bleeding, bruising, leakage, injection site reactions, usability, satisfaction, convenience, fear, and adherence. Most studies reported lower pain frequency, fewer injection site reactions, less bleeding or bruising, or improved patient preference with smaller-diameter needles.

The most consistent finding was a reduction in the frequency of painful injections. In healthy volunteers, Arendt-Nielsen et al., Egekvist et al., and Wägo et al. reported less frequently painful injections with smaller-diameter needles, although differences in pain intensity measured using VAS were less consistent. (Egekvist et al. 1999; Arendt-Nielsen et al. 2006; Wågø et al. 2016) Several studies in patients with diabetes similarly found lower pain, less fear, reduced bleeding or bruising, or greater satisfaction with smaller-diameter needles (Iwanaga and Kamoi 2009; McKay et al. 2009; Miyakoshi et al. 2007). Fear was unfortunately measured with a VAS rather than a validated instrument. (Miyakoshi et al. 2007) In patients with multiple sclerosis, most respondents reported that a 29 G needle was less painful and associated with fewer injection site reactions than a 27 G needle. (Glenski 2009) However, findings were not uniform. Hanas et al. found no significant differences in pain between needles ranging from 27 to 30 G despite less frequent bleeding with a 29 G needle compared with a 28 G needle, (Hanas et al. 2000) while Boyd et al., Robb and Kanji, Præstmark et al., and van Dommelen et al. reported either non-significant trends or no association between needle diameter and outcomes assessed (Robb and Kanji 2002; Boyd et al. 2008; Præstmark et al. 2016; van Dommelen et al. 2024).

Interpretation is limited because many studies compared needles that differed simultaneously in diameter, length, tapering, bevel design, wall structure, or device configuration. For example, Jaber et al. compared needles that differed in both gauge and bevel design, (Jaber et al. 2008) while Iwanaga and Kamoi, Miyakoshi et al., and Nagai et al. compared needles that differed in both gauge and tapering. (Miyakoshi et al. 2007; Iwanaga and Kamoi 2009; Nagai et al. 2013) Chan et al. compared needles that also differed in length and design. (Chan et al. 2004) Consequently, although the overall direction of evidence favours smaller-diameter needles and pain variations are likely to be more noticeable between needles that vary more widely in gauge (e.g. 23 may be noticeably more painful than 30 compared with 27 vs. 30), the magnitude of benefit attributable specifically to needle diameter remains uncertain.

##### 3.4.1.3. Needle bevel/tip.

Five studies evaluated bevel number, bevel geometry, bevel orientation, or tip angle in 156 participants (**Supplementary Table 4**) (Omoigui et al. 2006; Gill et al. 2008; Jaber et al. 2008; Hirsch et al. 2012; Præstmark et al. 2016). Outcomes primarily included pain and acceptability. Overall, evidence suggested modest benefits of certain bevel designs, although findings were less consistent than those observed for needle diameter or length.

Studies comparing 5-bevel and 3-bevel needles generally favoured 5-bevel designs. Hirsch et al. found no significant difference during blinded testing, but reported lower pain, greater comfort, easier insertion, and greater preference for 5-bevel needles during home use (Hirsch et al. 2012). Jaber et al. similarly reported consistently lower pain scores with 5-bevel than 3-bevel needles (Hirsch et al. 2012). In contrast, Præstmark et al. found no clinically relevant differences in pain perception with a range of tip designs (1, 2, 3, or 5 bevels; asymmetrical tip; obtuse tip) in 30 patients receiving insulin injections (Præstmark et al. 2016).

Evidence for other tip characteristics was mixed. Gill et al. found no significant differences in pain between microneedles with different tip angles, although lower angles showed a trend toward lower pain for one microneedle length (Gill et al. 2008).

Overall, interpretation was limited because several comparisons differed in additional characteristics, including gauge and shield materials.

##### 3.4.1.4. Needle tapering.

Evidence from four randomized crossover studies in 248 patients with diabetes suggests that tapered needle designs may improve patient experience, although findings were not entirely consistent and tapering was invariably evaluated alongside other needle characteristics (**Supplementary Table 5**) (Asakura et al. 2006; Miyakoshi et al. 2007; Iwanaga and Kamoi 2009; Nagai et al. 2013).

Three of the studies reported less pain, reduced fear, less bruising or bleeding, and greater patient satisfaction with tapered needles compared with non-tapered comparators (Asakura et al. 2006; Miyakoshi et al. 2007; Iwanaga and Kamoi 2009). In contrast, Nagai et al. found that a shorter 4-mm straight needle was associated with significantly lower pain, less bleeding, and greater patient satisfaction than a longer microtapered needle (Nagai et al. 2013).

However, in all four studies, tapered and non-tapered needles differed not only in tapering but also in gauge, length, wall design, or multiple design characteristics simultaneously. For example, the tapered needle Iwanaga and Kamoi evaluated was both narrower at the tip and longer than the comparator, while the microtapered needle Nagai et al. assessed differed in gauge, length, and wall structure. Consequently, it is not possible to determine the independent effects of tapering on patient outcomes, which were furthermore measured with non-standardized VAS.

Taken together, the available evidence suggests that tapered needles are associated with improved patient outcomes, but these benefits may reflect a combination of needle design features rather than tapering alone.

##### 3.4.1.5. Needle wall thickness.

Three studies evaluated needle wall thickness (**Supplementary Table 6**) (Gill et al. 2008; Siegmund et al. 2009; Aronson et al. 2013). Overall, evidence favored thin- or extra-thin-wall designs, although the evidence base was significantly smaller than that for needle diameter or length.

Aronson et al. found that extra-thin-wall pen needles were preferred to standard-wall needles because they required less thumb force, increased confidence in complete dose delivery and ease and convenience of use, and decreased pain (Aronson et al. 2013). Similarly, Siegmund et al. found that patients reported less pain, bleeding, skin irritation, leakage, and needle occlusion with a thin-wall needle than with a regular-wall comparator (Siegmund et al. 2009). In contrast, Gill et al. found no significant association between microneedle wall thickness and pain (Gill et al. 2008). Overall, the available evidence suggests that thin-wall technologies may improve patient experience and injection performance, although the small number of studies and heterogeneity of outcomes limit confidence regarding the magnitude of the benefit.

##### 3.4.1.6. Microneedles.

Two studies evaluated microneedles in a total of 50 participants (**Supplementary Table 7**); (Gill et al. 2008; Lee et al. 2018) both reported lower pain than conventional hypodermic or pen needles. Gill et al. reported significantly less pain with microneedles of all lengths and widths evaluated in comparison with a 26 G hypodermic needle, with pain reductions of 60–95% (Gill et al. 2008). There were no differences in pain between the microneedles included in this study. Lee et al. similarly reported less pain and less injection site bleeding with a 4-mm microneedle than with a conventional 32 G pen needle (Lee et al. 2018). Although limited to two studies, the available evidence suggests that microneedles substantially reduce injection pain compared with conventional SC needles. However, evidence regarding anxiety, usability, adherence, or long-term patient preference was not reported.

##### 3.4.1.7. SC infusion needle characteristics.

Seven studies evaluated the effects of SC infusion needle characteristics in 266 participants, primarily in palliative care populations (**Supplementary Table 8**) (Youssef and Atkinson 1990; Currow and Cooney 1994; Macmillan et al. 1994; Dawkins et al. 2000; Chan et al. 2004; Abbas et al. 2005; Neo et al. 2016). Overall, plastic, Teflon, or polyurethane cannulas were consistently associated with fewer injection site reactions and improved patient experience compared with metal buttery needles, including less inflammation, swelling, erythema, tenderness, or other injection site reactions.

The most consistent benefit was a reduction in injection site reactions. In a crossover study of 54 patients with thalassemia, Chan et al. found significantly lower rates of pain, itching, tenderness, and swelling with an 8 mm Thalaset needle than with a 19 mm butterfly needle (Chan et al. 2004). Similarly, Dawkins et al. reported fewer injection site reactions and fewer needle re-siting events with plastic cannulas than with metal needles, (Dawkins et al. 2000) while Neo et al. observed pain, blistering, erythema, and swelling only among patients with metal needles (Neo et al. 2016).

Evidence regarding patient comfort and preference was more limited but favored plastic-based systems. Macmillan et al. found that patients preferred Teflon cannulas because of a reduced need for injection site changes, although no difference in comfort was observed between devices (Macmillan et al. 1994).

Interpretation of these findings is limited by the predominance of small studies conducted in palliative care settings and by comparisons that often differed simultaneously in needle material, gauge, length, and device design. Nevertheless, the direction of evidence was notably consistent, with no study reporting superior outcomes for metal needles.

#### 3.4.2. Device-related factors

##### 3.4.2.1. Device choice.

Eight studies involving 1,847 participants compared autoinjectors with prefilled syringes or conventional vial-and-syringe administration across diabetes, rheumatoid arthritis, chronic kidney disease, and healthy volunteer populations (**Supplementary Table 9**) (Kadiri et al. 1998; Kivitz et al. 2006; Borrás-Blasco et al. 2010; Lim et al. 2012; Ramael et al. 2018; Saraux et al. 2019; Alten et al. 2020; Zhang et al. 2021). Outcomes included pain, patient preference, stress, usability, adherence, and injection site reactions.

Overall, the evidence favoured autoinjectors. Most studies reported lower pain scores, (Kadiri et al. 1998; Kivitz et al. 2006; Borrás-Blasco et al. 2010; Lim et al. 2012; Alten et al. 2020) greater ease of use, or stronger patient preference (Lim et al. 2012) with autoinjectors than with prefilled syringes or conventional vial-and-syringe administration. For example, Alten et al. reported lower injection site pain with an autoinjector than with either a prefilled syringe or vial/syringe, and lower with a prefilled syringe than with a vial/syringe (Alten et al. 2020).

However, findings were not uniform. Lim et al. found no significant difference in pain despite a majority of patients preferring the autoinjector (Lim et al. 2012). Similarly, Saraux et al. observed only small differences in pain and stress between devices (Saraux et al. 2019). One study found a higher rate of injection site reactions with an autoinjector compared with a prefilled syringe (Zhang et al. 2021).

Interpretation is limited because autoinjectors differ from comparator devices in multiple respects, including needle concealment, ergonomics, injection mechanics, and delivery speed. Consequently, it is not possible to determine which individual device characteristics were primarily responsible for the observed benefits.

##### 3.4.2.2. Hidden/retractable needle.

Five studies evaluated hidden or retractable needle systems in 644 participants (**Supplementary Table 10**) (Lamblet et al. 2011; D'Arcy et al. 2012; Devonshire et al. 2016; Al Hayek and Al Dawish 2021; Pivato et al. 2024). Outcomes included fear/anxiety, usability, pain, bruising, bleeding, device preference, and injection-related adverse events. Overall, the evidence favored hidden or retractable needle systems for fear-related and usability outcomes.

Al Hayek et al. reported significant reductions in all assessed dimensions of fear of self-injection, as well as reductions in pain, bleeding, bruising, and accidental needle-stick injuries when a safety pen needle was used instead of a conventional needle (Al Hayek and Al Dawish 2021). However, while fear decreased and a validated measure was used, the single-arm pre/post design prevents independent attribution to concealment alone. Devonshire et al. reported reductions in pre-injection anxiety and injection fear following use of a hidden-needle autoinjector, (Devonshire et al. 2016) while D’Arcy et al. found that needle concealment was ranked as the most useful feature of the evaluated autoinjector (D'Arcy et al. 2012). Pivato et al. found that hidden needle visibility contributed to preference for prefilled pens over syringes (Pivato et al. 2024). Finally, Lamblet et al. reported that a retractable needle resulted in significantly less bruising; while pain was also measured in this study, intramuscular and subcutaneous injections were combined and could not be distinguished (Lamblet et al. 2011).

However, all studies evaluated needle concealment as part of a broader delivery system. Consequently, it was not possible to determine whether improvements were attributable specifically to concealment or to other device characteristics such as autoinjection, ergonomics, training, or ease of use.

#### 3.4.3. Administration-related factors

Four studies evaluated aspects of injection administration in 615 participants (**Supplementary Table 11**) (Egekvist et al. 1999; Candiotti et al. 2009; Müller-Ladner et al. 2010; Striesow and Brandt 2012). Outcomes included pain, patient preference, and usability. Candiotti et al. found that administration of lidocaine with the needle bevel up rather than down was significantly associated with decreased patient pain (Candiotti et al. 2009). Egekvist et al. reported that needle insertion velocity influenced pain quality, with faster insertion associated with more sharp and pricking pain and slower insertion velocity associated with more dull pain, despite no overall difference in pain occurrence (Egekvist et al. 1999). This study also found no difference in pain occurrence between a 45° or 90° angle of insertion. Two studies evaluated methotrexate administration systems incorporating pre-attached needles (Müller-Ladner et al. 2010; Striesow and Brandt 2012). Müller-Ladner et al. reported that most patients considered the pre-attached needle an advantage; (Müller-Ladner et al. 2010) unfortunately, due to the study design, it was not possible to determine the individual impact of the needle design independent from the syringe size or the formulation. A subsequent post-marketing surveillance study found similarly favourable patient perceptions (Striesow and Brandt 2012). Overall, the available evidence suggests that administration technique and delivery system configuration may influence patient experience independently of needle design, although the evidence base remains limited and highly heterogeneous.

### 3.5. Qualitative assessment of certainty of evidence

Qualitative assessment of certainty of evidence suggested that confidence varied across outcomes (**Supplementary Table 15**). Pain outcomes were supported by the largest body of evidence and demonstrated consistent directional findings across randomized studies; however, confidence was moderated by the predominance of open-label designs, subjective outcome assessments, and frequent concerns regarding selective reporting. Confidence in evidence anxiety and fear outcomes was limited because these outcomes were assessed in few studies, validated instruments were used in only two studies, and many studies relied on subjective or non-validated measures and had serious risk of bias. Confidence in injection site safety outcomes, including bleeding, bruising, leakage, and injection site reactions, was considered limited because outcome definitions, ascertainment methods, and comparator interventions varied across studies. Confidence in adherence, usability, satisfaction, and preference outcomes was also limited because these outcomes were often assessed using subjective measures in unblinded studies.

## 4. Discussion

This systematic review synthesized findings from 62 studies to explore how SC needle characteristics and device- and administration-related factors influence patient-reported pain, anxiety, and safety. A key consideration throughout the interpretation of this body of literature is the distinction between needle-specific attributes such as gauge, length, bevel design, wall thickness, and lubricity and broader device- and administration-related characteristics such as autoinjectors, PFSs, OBIs, hidden or retractable systems, injection speed, and pressure-sensitive delivery mechanisms. However, many included studies assessed combinations of these factors, which limits the ability to isolate effects attributable specifically to needle design. Overall, the evidence base was heterogeneous but was most consistent for patient-reported pain and selected local injection site safety outcomes, where thinner gauge, shorter length, and certain bevel geometries appear to be associated with improved tolerability. Anxiety and fear were less frequently evaluated, but the available evidence generally favoured concealed, (Devonshire et al. 2016; Al Hayek and Al Dawish 2021) shorter, (Bergenstal et al. 2015; Pager et al. 2020) or thinner (Miyakoshi et al. 2007; Iwanaga and Kamoi 2009) needles. However, even within these domains, attribution is often confounded by concurrent differences in device design or administration method.

Higher needle gauge (i.e. thinner needles) was most consistently associated with lower pain intensity, bleeding, bruising, and patient discomfort. Multiple studies demonstrated that 32 G needles were associated with lower pain scores and greater patient preference compared with thicker alternatives on VAS and preference measurements, including small incremental differences such as between 31 G and 32 G (Iwanaga and Kamoi 2009) or with 1-mm differences in needle length (Nagai et al. 2013). One study found pain-free injection in 24% of patients using a 32 G needle versus 15% using a 30 G needle (McKay et al. 2009). However, other studies found numerical differences but not statistically significant differences, which may reflect small sample sizes, parallel-group designs rather than crossover methods, subjective pain assessments, or simultaneous variation in multiple needle characteristics (e.g. gauge and length) or confounding by device and administration factors, including differences in injection system, speed, and technique. In crossover designs, where patients act as their own controls, variability in subjective pain reporting may be reduced, although risk of bias remains due to non-blinding and expectation effects.

For manual injections of viscous large-volume SC biologics such as those coformulated with hyaluronidase at high concentrations, larger diameters (e.g. 23-25 G) and more injection force are required to ensure adequate flow rates (Jaber et al. 2008; Strickley and Lambert 2021; Desai et al. 2023; Desai et al. 2024; Kang et al. 2025). These parameters reflect an interaction between needle properties (gauge and internal diameter) and biologic-related characteristics (viscosity, volume, and formulation properties), which together determine flow dynamics. While thinner needles are preferred by patients, this efficiency may come at the cost of patient comfort, as patients appear to prefer thinner needles and slower injection speeds (Ramael et al. 2018; van Dommelen et al. 2024). Automatic needle insertion systems and device-assisted delivery reduce variability in administration technique and perceived pain (Jørgensen 1994).

**Figure 6.**
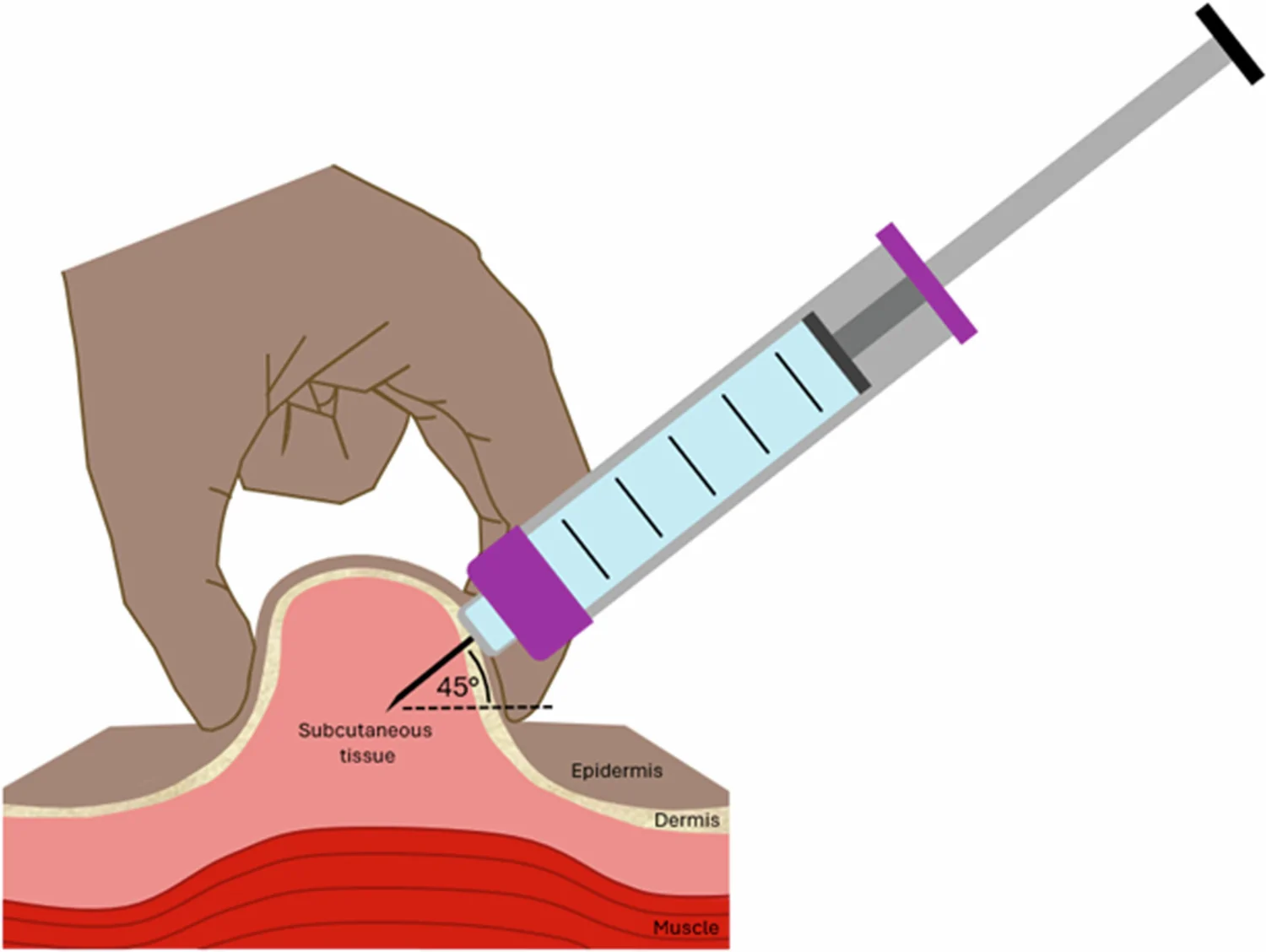
Skin Pinch-Up Method.

Shorter needles were generally associated with decreased pain and adverse events, particularly among patients with diabetes. However, interpretation is limited by confounding between needle length, gauge, and administration technique (e.g. skin pinch-up; Figure 6), complicating attribution of effects. Microneedles and extra-thin wall needles showed promise in reducing penetration force and injection trauma, (Præstmark et al. 2016) but real-world data remain limited.

Bevel and tip geometry also influenced insertion comfort and reduced pain in some studies, with five-bevel needles generally outperforming three-bevel alternatives (Figure 1). These features are more directly attributable to needle-specific characteristics, although interpretation remains limited where bevel design is confounded by gauge, lubrication, device integration, or shield material. Some evidence suggests that gauge may be a more influential determinant of cutting force and pain than bevel geometry alone, (Mayer et al. 2009) although further controlled studies are needed.

Evidence on needle dulling is also limited. Dulling after repeated puncture increased penetration force in several studies, (Majcher et al. 2018) and septum passage increased resistance modestly, although brand and gauge had greater effects (Tawil et al. 2025). Bevel angle appears to affect needle durability, with a 19° back-cut needle more resistant to deterioration than 15° or 17° designs (Urimoto et al. 2024).

Anxiety outcomes were less systematically characterized than pain outcomes. Only two studies used validated anxiety or fear instruments (Diabetes Fear of Injecting and Self-Testing Questionnaire [D-FISQ] and the Hospital Anxiety and Depression Scale—Anxiety subscale [HADS-A]/State-Trait Anxiety Inventory [STAI]), (Devonshire et al. 2016; Al Hayek and Al Dawish 2021) whereas most relied on study-specific questionnaires or VAS ratings of frightening needle appearance or stress during injection (Schwartz et al. 2004; Miyakoshi et al. 2007; Iwanaga and Kamoi 2009; Bergenstal et al. 2015; Saraux et al. 2019; Pager et al. 2020). Timing of assessment varied substantially, ranging from anticipatory ratings before simulated injection and baseline needle phobia assessments to evaluations after weeks or months of device use (Hanas et al. 2000; Lim et al. 2012; Bergenstal et al. 2015; Devonshire et al. 2016; Saraux et al. 2019; Pager et al. 2020). Most studies were open-label; the only double-blind study measured needle phobia at baseline and did not assess intervention-related changes (Hanas et al. 2000). Three of the seven studies found no significant differences in fear, anxiety, or stress (Schwartz et al. 2004; Lim et al. 2012; Saraux et al. 2019). Although concealed-needle devices were generally associated with reduced fear or greater reassurance, available studies did not sufficiently control for device type, self- vs. HCP-administration, training, novelty, or repeated-exposure effects, limiting causal inference regarding anxiety reduction.

Interpretation of the findings should consider the varying certainty of evidence across outcomes. The strongest evidence was available for pain, which was the most frequently assessed endpoint and generally demonstrated consistent directional findings across multiple randomized studies. However, confidence in these findings was tempered by the predominance of open-label study designs and reliance on subjective patient-reported measures. Confidence in conclusions relating to anxiety and fear was limited because relatively few studies evaluated these outcomes, validated anxiety instruments were used in only two studies, and most contributing studies were judged to have serious risk of bias. Similarly, confidence in injection site safety outcomes was limited by heterogeneity in outcome definitions and reporting practices. Evidence relating to adherence, usability, satisfaction, and patient preference should likewise be interpreted cautiously because these outcomes were frequently subjective and vulnerable to expectation, novelty, and performance biases.

Limitations of the included studies were substantial and included heterogeneity in populations, designs, and outcome measures. Studies varied widely in population (pediatric vs. adult, healthy vs. chronically ill, treatment-experienced vs. treatment-naïve), design (crossover vs. parallel-group), and outcome measures (VAS, subjective ratings, differing definitions of adverse events). Newly diagnosed or treatment-naïve patients may experience more fear and anxiety about injection-related pain and more perceived pain, (Kruger et al. 2015; Mekala et al. 2021; Kazum et al. 2024) while high-burden populations (e.g. patients with diabetes) may experience more ongoing, low-level pain, cumulative irritation, and residual discomfort (St Clair-Jones et al. 2020). Some studies reported differences in pain frequency but not intensity, underscoring the importance of standardized, multi-dimensional pain measurement. Pain outcomes were assessed using a wide variety of instruments and scale formats, including visual analogue scales with different ranges and anchors, categorical ratings, preference measures, and non-standardized assessments. This variability limited direct comparison across studies and precluded meaningful normalization or transformation of pain scores across the evidence base. Although rescaling approaches and standardized effect size calculations can facilitate comparisons in some settings, the substantial differences in outcome constructs, assessment timing, reporting practices, and availability of statistical data limited their applicability in the present review. This heterogeneity limited the ability to perform robust meta-analyses and generalize results across clinical settings. Many studies assessed multiple needle or device attributes simultaneously, limiting causal inference and making it difficult to disentangle needle-specific effects from device- and administration-related influences. Psychological outcomes relied heavily on subjective measures without use of validated anxiety tools, increasing risk of measurement bias and variability and hindering meaningful comparison across studies. More broadly, reporting of patient-reported outcomes was inconsistent across studies, with substantial variation in outcome definitions, measurement approaches, and reporting detail. Future studies would benefit from greater use of standardized patient-reported outcome methodologies and reporting frameworks to improve interpretability and comparability. Safety outcomes were similarly heterogeneous. Definitions and reporting of bleeding, bruising, leakage, erythema, infection, and other injection-site events varied substantially across studies, and many safety endpoints occurred infrequently. Several studies were not designed or powered to detect differences in uncommon adverse events, limiting confidence in comparative safety conclusions. Consequently, the apparent absence of differences for some safety outcomes should not necessarily be interpreted as evidence of equivalence. The heterogeneity of interventions, comparators, outcome measures, and reporting practices also limited the utility of formal vote-counting approaches based solely on direction of effect. In many studies, multiple needle characteristics, device features, and administration-related variables were evaluated simultaneously, making assignment of a single direction-of-effect potentially oversimplistic and susceptible to misinterpretation. Finally, risk of bias was high or unclear in many studies due to lack of blinding, inadequate randomization, and small sample sizes.

This systematic review was also subject to several methodological limitations, including restriction to English-language publications, which may introduce language bias. The review protocol was not prospectively registered in PROSPERO, which may reduce transparency and increase the risk of unintentional methodological deviations or selective reporting; however, predefined review methods were consistently applied throughout the study process to support methodological rigor and minimize bias. The risk of bias assessment was conducted by a single reviewer, which may increase susceptibility to subjective judgment and inconsistency; however, predefined assessment criteria and structured review procedures were used to promote consistency and minimize bias in the evaluation process. In addition, while the inclusion of observational study designs may introduce variability in methodological quality, their inclusion allowed for a more comprehensive synthesis of the available evidence in an area where higher-level study designs remain limited. Finally, the ability to evaluate needle-specific effects independently of device-, administration-, and biologic-related factors was limited. Relevant subgroup analyses by indication, device class, injection site, and biologic properties (e.g. viscosity, volume, flow rate, pH, and excipient composition) were constrained by inconsistent reporting and substantial heterogeneity across studies. Consequently, some observed differences may reflect interactions between needle characteristics and broader delivery-system or formulation factors rather than needle design alone.

Despite these limitations, several practical implications emerged. Needle-specific characteristics, including thinner gauge, shorter length, tapered bevels, and hidden or lubricated designs, may enhance patient comfort and reduce adverse events. These effects are most plausibly attributable to reduced penetration force and tissue trauma. Hidden needles may benefit patients with needle anxiety, such as adolescents or patients with chronic self-injection requirements. Tailoring needle technologies to minimize pain and anxiety may support better adherence, especially in long-term therapies (Norman and Prausnitz 2012; Kruger et al. 2015; Præstmark et al. 2016; Gandell et al. 2019; Antalfy et al. 2023).

Injection technique also represents an important administration-related factor (Figure 6). The skin pinch-up method may reduce pain related to longer needle lengths (Strauss et al. 1999) but is subject to user variability. Some OBIs automate this in a consistent manner via tissue-tenting features. (Huddleston 2025) Patients may also tolerate longer needles if injections are slower (van Dommelen et al. 2024). Similarly, injection speed and delivery dynamics are likely important determinants of injection site reactions (van Dommelen et al. 2024). Evidence generally suggests fewer injection site reactions with autoinjectors than with PFSs, although findings are not uniform.

More advanced device- and administration-level technologies, such as pressure-sensitive delivery systems, are increasingly relevant in the context of SC injection, where tissue counterpressure increases with viscosity, rate, and volume (Allmendinger et al. 2015). Real-time pressure-sensing may enable adaptive flow modulation, slowing delivery to improve dose accuracy and reduce discomfort (Allmendinger et al. 2015). OBIs may also reduce injection site reactions (Wasserman et al. 2022) and pain (Quach et al. 2023) with consistent needle administration and adaptive pressure-based delivery; however, these effects are attributable to integrated device systems rather than needle characteristics alone. Reviews of large-volume SC platforms further support the role of controlled injection rates and pressure/occlusion feedback systems in improving user experience, adherence, and delivery reliability. (Antalfy et al. 2023).

From a development perspective, these findings underscore the importance of human-factors engineering and patient-centered design. For example, pen needle hub optimization reduced penetration depth variability and decreased intramuscular injection regardless of differences in user techniques in one study, (Rini et al. 2022) and iterative user feedback improved the design of an electromechanical certolizumab pegol injector in another (Domańska et al. 2018). Patient-centered features may also support adherence during treatment initiation (Boeri et al. 2019).

Future research should prioritize the isolation of individual needle characteristics using controlled factorial designs; separately evaluate device- and administration-related variables such as injection speed, system type, and delivery mechanics; use standardized, validated measures of pain, anxiety, and safety; employ crossover designs; include diverse populations; compare advanced needle designs (e.g. microneedles, retractable systems) in real-world, longitudinal settings; and assess economic and adherence outcomes. Large, randomized trials and real-world studies are needed to evaluate the long-term benefits of needle optimization on clinical outcomes and patient satisfaction.

## 5. Conclusion

This systematic review found that SC needle characteristics, particularly gauge, length, bevel geometry, wall thickness, and concealment, are associated most consistently with patient-reported pain and selected local injection site safety outcomes. The evidence suggests that thinner, shorter, tapered, and hidden needles may improve injection comfort and reduce minor adverse events. These improvements may support adherence, reduce treatment fatigue, decrease healthcare resource use, and improve clinical outcomes, especially in chronic treatment. However, variability across studies underscores the need for standardized methods and direct evaluation of individual needle attributes. Overall, while needle design appears to influence patient experience during SC administration, the strength of the evidence varies considerably across outcomes. More robust, standardized, and mechanistically focused studies are required to clarify the independent effects of specific needle characteristics and to determine their true impact on broader behavioral, economic, and clinical outcomes.

## Supplementary Material

Needles SLR Supplementary Info.docx Needles SLR Supplementary Info.docx

Needles SLR PRISMA_2020_checklist.pdf Needles SLR PRISMA_2020_checklist.pdf

## Data Availability

Data used to develop this review are available from the corresponding author upon request.
