# Supplementary material for: Analyzing the impact of subcutaneous injection needle, device, and administration characteristics on patient pain, anxiety, and safety: a systematic literature review
Source: Drug Deliv. 2026 Aug 27;33(1):2719057. doi: 10.1080/10717544.2026.2719057 (PMC13523121; doi:10.1080/10717544.2026.2719057)
Supplement: Supplementary file 2 [file IDRD_A_2719057_SM5295.docx]

**Supplemetary Information**

**Supplementary Table 1: Protocol**

| **PubMed/Medline Search Strategy** | (("subcutaneous"[All Fields] OR "subcutaneously"[All Fields] OR "subcutaneous"[All Fields]) AND ("needles"[All Fields] OR "needled"[All Fields] OR "needles"[MeSH Terms] OR "needle"[All Fields] OR "needlings"[All Fields] OR "percutaneous collagen induction"[MeSH Terms] OR ("percutaneous"[All Fields] AND "collagen"[All Fields] AND "induction"[All Fields]) OR "percutaneous collagen induction"[All Fields] OR "needling"[All Fields]) AND ("anxiety"[MeSH Terms] OR "anxiety"[All Fields] OR "anxieties"[All Fields] OR "anxiety s"[All Fields] OR ("fear"[MeSH Terms] OR "fear"[All Fields]) OR ("pain"[MeSH Terms] OR "pain"[All Fields]) OR ("phobic disorders"[MeSH Terms] OR ("phobic"[All Fields] AND "disorders"[All Fields]) OR "phobic disorders"[All Fields] OR "phobia"[All Fields] OR "phobias"[All Fields]) OR ("safety"[MeSH Terms] OR "safety"[All Fields] OR "safeties"[All Fields]) OR ("injection site reaction"[MeSH Terms] OR ("injection"[All Fields] AND "site"[All Fields] AND "reaction"[All Fields]) OR "injection site reaction"[All Fields]) OR ("efficacies"[All Fields] OR "efficacious"[All Fields] OR "efficaciously"[All Fields] OR "efficaciousness"[All Fields] OR "efficacy"[All Fields]) OR ("patient compliance"[MeSH Terms] OR ("patient"[All Fields] AND "compliance"[All Fields]) OR "patient compliance"[All Fields] OR ("patient"[All Fields] AND "adherence"[All Fields]) OR "patient adherence"[All Fields])) AND ("humans"[MeSH Terms] AND "english"[Language])) AND ((booksdocs[Filter] OR clinicalconference[Filter] OR clinicalstudy[Filter] OR clinicaltrial[Filter] OR clinicaltrialphasei[Filter] OR clinicaltrialphaseii[Filter] OR clinicaltrialphaseiii[Filter] OR clinicaltrialphaseiv[Filter] OR comparativestudy[Filter] OR congress[Filter] OR controlledclinicaltrial[Filter] OR correctedandrepublishedarticle[Filter] OR editorial[Filter] OR englishabstract[Filter] OR evaluationstudy[Filter] OR multicenterstudy[Filter] OR observationalstudy[Filter] OR pragmaticclinicaltrial[Filter] OR randomizedcontrolledtrial[Filter] OR twinstudy[Filter] OR validationstudy[Filter])) |
| --- | --- |
| **Embase Search Strategy** | ('subcutaneous':ti,ab,de OR 'subcutaneously':ti,ab,de OR 'subcutanous':ti,ab,de) AND ('needles':ti,ab,de OR 'needled':ti,ab,de OR 'needle'/exp OR 'needle' OR 'needle':ti,ab,de OR 'needlings':ti,ab,de OR 'microneedling'/exp OR 'microneedling' OR ('percutaneous':ti,ab,de AND 'collagen':ti,ab,de AND 'induction':ti,ab,de) OR 'percutaneous collagen induction':ti,ab,de OR 'needling':ti,ab,de) AND ('anxiety'/exp OR 'anxiety' OR 'anxiety':ti,ab,de OR 'anxieties':ti,ab,de OR 'anxiety s':ti,ab,de OR 'fear'/exp OR 'fear' OR 'fear':ti,ab,de OR 'pain'/exp OR 'pain' OR 'pain':ti,ab,de OR 'phobia'/exp OR 'phobia' OR ('phobic':ti,ab,de AND 'disorders':ti,ab,de) OR 'phobic disorders':ti,ab,de OR 'phobia':ti,ab,de OR 'phobias':ti,ab,de OR 'safety'/exp OR 'safety' OR 'safety':ti,ab,de OR 'safeties':ti,ab,de OR 'injection site reaction'/exp OR 'injection site reaction' OR ('injection':ti,ab,de AND 'site':ti,ab,de AND 'reaction':ti,ab,de) OR 'injection site reaction':ti,ab,de OR 'efficacies':ti,ab,de OR 'efficacious':ti,ab,de OR 'efficaciously':ti,ab,de OR 'efficaciousness':ti,ab,de OR 'efficacy':ti,ab,de OR 'patient compliance'/exp OR 'patient compliance' OR 'treatment refusal'/exp OR 'treatment refusal' OR ('patient':ti,ab,de AND 'compliance':ti,ab,de) OR 'patient compliance':ti,ab,de OR ('patient':ti,ab,de AND 'adherence':ti,ab,de) OR 'patient adherence':ti,ab,de) AND [english]/lim AND [humans]/lim AND ('article'/it OR 'article in press'/it OR 'chapter'/it OR 'conference abstract'/it OR 'conference paper'/it OR 'conference review'/it OR 'editorial'/it OR 'letter'/it OR 'note'/it OR 'preprint'/it OR 'short survey'/it) |
| **ClinicalTrials.gov Search Strategy** | Other terms: Needle  Intervention/treatment: “Subcutaneous Injection” |
| **STUDY RECORDS** | |
| **Study Selection** | Databases will be searched from inception to the date of the search, and search alerts will be put in place to collect any additional records published during the systematic review and manuscript preparation process. Two individuals will independently screen all studies retrieved in searches and will remove duplicates and then exclude studies based first on title, then abstract, then full-text. Reference lists from selected articles will also be screened. Any differences in the screening results will be discussed in order to reach a consensus regarding full-text article inclusion in the review. After both individuals have determined the full-text articles eligible for inclusion, both individuals will use a semi-structured data collection form to participate in manual data extraction. Where necessary and feasible, attempts will be made to contact the authors of included studies for any missing information or clarification required. |
| **Data Extraction** | The data detailed below will be initially collected in an Excel file for use in the meta-analysis. Any discrepancies in the data extracted will be evaluated and addressed.   - Title of publication - Author(s) - Year of publication - Title of journal - PICO: population (patients or healthy volunteers), intervention (subcutaneous injection), comparison (differing needle characteristics, e.g., diameter, hidden/retractable mechanism, bevel/tip, length, wall width, administration/insertion), outcome (anxiety, fear, pain, safety, and/or injection efficacy) - Participant inclusion and exclusion criteria - Study methods and setting(s) - Population and participant characteristics - Intervention description(s) - Outcome measure(s) - Results - Number of and reasons for withdrawals and/or dropouts |
| **Risk of Bias in Individual Studies** | Risk of bias in individual studies will be evaluated with the Revised Cochrane risk-of-bias tool for randomized trials (RoB 2), the Risk Of Bias In Non-randomized Studies - of Interventions (ROBINS-I) tool for non-randomized interventional studies, the Newcastle-Ottawa Scale (NOS) for cohort and case-control studies, and the Joanna Briggs Institute (JBI) critical appraisal checklist for cross-sectional studies. |
| **DATA** | |
| **Synthesis** | If the number of studies and quality of the data allows, a meta-analysis will be conducted to determine the effects of needle characteristics on anxiety, fear, pain, safety, and/or injection efficacy in individuals receiving subcutaneous injections. Odds ratios and/or standardized mean differences and corresponding confidence intervals will be calculated to evaluate the relationships between needle characteristics and outcomes with significance set at p < 0.05. Heterogeneity among studies will be examined using Q-test statistics with significance set at p < 0.01 and I^2^ statistics with significance set at > 50%. Fixed effects models will be used except where significant heterogeneity is found, in which case random effects models will be used to calculate pooled effect sizes. |
| **Analysis of Subgroups or Subsets** | If there are sufficient data for subgroup analysis, the meta-analysis will include analyses of experienced (e.g., 3+ years since diagnosis of condition requiring subcutaneous injections) vs. inexperienced (e.g., < 3 years since diagnosis of condition requiring subcutaneous injections) patients, adults vs. children and adolescents, and by injection location (e.g., abdomen vs. thigh). |
| **Meta-bias(es)** | Publication bias will be assessed with a funnel plot and Egger’s test for asymmetry with significance set at p < 0.05. |
| **Confidence in Cumulative Evidence** | The Grading of Recommendations, Assessment, Development, and Evaluations (GRADE) framework will be used to determine confidence in the quality of the cumulative evidence. |

**Supplementary Table 2: Summary of Articles Assessing the Impact of Needle Length on Pain, Anxiety, and Safety**

| **Reference** | **Study Design** | **N** | **Indication** | **Needle Characteristics** | **Outcome Measures** | **Key Findings** |
| --- | --- | --- | --- | --- | --- | --- |
| Al Hayek et al., 2021b^87^ | Prospective cohort study | 62 | Type 1 diabetes | 6-mm and 8-mm 32.5 G | VAS pain score, Morisky Medication Adherence Scale, needle attribute score, injection-related adverse events | 6-mm needle was associated with lower pain score, higher adherence, and fewer adverse events than the 8-mm needle |
| Berard et al., 2015^109^ | Sub-study of single-site, open-label, randomized, comparative study | 66 | Type 2 diabetes | 8-mm pen needle for first three months, 5-mm pen needle for second three months | Questionnaire about pen needle satisfaction | 5-mm needle was preferred over 8-mm needle and was considered more comfortable and easier to use |
| Bergenstal et al., 2015^90^ | Prospective, multicenter, randomized, open-label, two-period, crossover, equivalence, home-based study | 274 | Diabetes | - 4-mm 32 G - 8-mm 31 G - 12.7-mm 29 G | Relative injection pain on a 150-mm VAS (0=as painful as the previous needle, +75=much more painful, -75=much less painful), patient diary entries about insulin leakage, number of pen needles returned vs. dispensed based on reported insulin therapy regimen to measure adherence | - 4-mm needles were preferred and less painful than 8- or 12.7-mm pen needles, were reported to be easier to use, easier to insert, and associated with less needle anxiety, and had equivalent insulin leakage and adverse events - 4-mm needle was reported to be easier for overall performance of injection and overall comfort compared with 12.7-mm needle - Adherence was 97%, 96%, and 96% for 4-, 8-, and 12.7-mm needle respectively |
| Chan et al., 2004^128^ | Intrapersonal crossover study | 54 | Transfusion-dependent thalassemia major treated with SC iron chelation for ≥2 years | - 27 G, 8-mm Thalaset needle - 25 G, 19-mm butterfly needle | Questionnaire in Chinese modified from common, existing pain analogue scale and simple quality of life assessment tools | Thalaset needle associated with fewer local reactions (e.g., pain, itchiness, tenderness, swelling) and reduced needle-related trauma |
| Coley et al., 1987^121^ | Prospective, randomized, single-blind | 73 | Coronary artery bypass graft surgery | - 25 G, 15.9-mm tuberculin syringe - 28 G, 12.7-mm insulin syringe | Clinical assessments, patient ratings of pain severity at injection | Tuberculin syringe was associated with significantly more pain and more severe pain compared with insulin syringe and no significant difference in hematoma |
| Dang et al., 2024^75^ | Single-center, randomized, single-blind, crossover study | 62 | Healthy volunteers | 6-, 9-, and 12-mm needles administered with syringe pump | Pain VAS, patient questionnaire about injection experiences and preferences | - No clinically meaningful differences in pain or participant preference between needle lengths - 12-mm needle associated with more injection site reactions than shorter needles |
| Gill et al., 2008^77^ | Randomized, double-blind | 10 | Healthy volunteers | Microneedle lengths of 480, 700, 960 and 1450 μm | Pain intensity on a 100-mm VAS | - Mean pain scores increased with increasing microneedle length (p=0.03) - Frequency of painless insertions (VAS score of 0) decreased with an increase in microneedle length (30% of patients with the 480 μm and 700 μm lengths vs. 0% and 10% with the 960 μm and 1450 μm microneedles) - Normalized data revealed increasing microneedle length increased pain, with a 3-fold increase in microneedle length from 480 μm to 1450 μm causing a >7-fold pain increase from 5% to 37% (corrected lower bound p = 0.013) |
| Hirsch et al., 2010;^93^ Hirsch et al., 2012b^94^ | Randomized, 4-center, non-inferiority crossover trial (2010); post-hoc analysis (2012) | 168 | Diabetes | - 32 G 4 mm - 31 G 5mm - 31 G 8 mm | Pain VAS and leakage at injection sites | Initial study   - 4-mm needle rated as significantly less painful and preferred by about 2/3 of patients (p<0.01) - No significant differences in injection site leakage   Post-hoc analysis   - 4-mm needle less painful than 5-mm needle in patients with obesity, and less painful than 8-mm needle in patients with and without obesity - Fewer leakage events with 4-mm needle compared to 5- or 8-mm needle in patients with and without obesity |
| Ignaut & Fu, 2009^95^ | Single-blind, randomized, two-period, crossover study | 56 | Diabetes | - 5 mm - 8 mm | Visual Analog Box-21 Scale for Pain, filter paper and an analytical balance for leakage, clinical assessment of bleeding and bruising | No significant difference in pain score, median leakage, proportion of injections with bleeding or bruising at injection site, or mean bruise size at injection site |
| Iwanaga & Kamoi, 2009^96^ | Randomized, open-label, crossover study | 30 | Diabetes | - 32 G 6-mm tapered needle and - 31 G 5-mm standard needle | 14-item questionnaire assessing overall satisfaction and usability of needles scored on a VAS from -100 (worst) to +100 (best) | Patients were more satisfied with the 32 G needle, found it less frightening, less painful, and reported that it caused less frequent bleeding and bruising than the 31 G needle |
| Kreugel et al., 2007^98^ | Crossover study | 68 | Diabetes | 5-, 8-, or 12-mm needle | VAS for pain, semi-qualitative questionnaire for injection-related side effects | 5-mm needle was associated with significantly less bleeding, bruising, and pain, had no significant difference in insulin leakage or frequency or severity of hypoglycemic events, and was significantly preferred to the longer needles |
| Kreugel et al., 2011^99^ | Multicenter, open-label, crossover study | 130 | Diabetes | 5- and 8-mm pen needles | 100-mm VAS for pain (0=no pain, 100=worst possible pain), validated questionnaire for injection-related side effects | - Less bleeding with 5-mm needle - Less insulin backflow with 8-mm needle - No significant difference in pain, bruising, or patient preference |
| Masid et al., 2015^112^ | Prospective | 38 | Multiple sclerosis | 4 or 6 mm for SC tissue thickness <25 mm, 6 or 8 for SC tissue thickness 25-50 mm, 8 or 10 mm for SC tissue thickness >50 mm | VAS for injection pain | No significant differences in pain on injection between different injection depths at any time point evaluated |
| McKay et al., 2009^100^ | Randomized, open-label, two-period, crossover trial | 119 | Diabetes | - 32 G 6-mm - 30 G 8-mm | Questionnaire evaluating needle preference and perceptions of pain, handling, an acceptance | - 58% preferred the shorter needle, 26% preferred the longer needle, and 16% had no preference (p<0.001 between needles) - Patients injecting more or fewer than 40 IU/day and more or fewer than two injections/day all preferred the shorter needle over the longer needle (all p<0.05) - 57% experienced little or no pain when using the shorter needle and 40% experienced little or no pain when using the longer needle - More patients found the injection button easier to press with the longer needle (p<0.01) - Ease of insertion, needle sharpness, and performance were rated higher for the shorter needle - No significant differences in bleeding or bruising at the injection site |
| Miwa et al., 2012^101^ | Prospective, open-label, controlled crossover study | 41 | Diabetes | - 32 G x 4 mm pen needle - 32 G 6 mm tapered pen needle | - Pain on a 150-mm VAS - Preference questionnaire with each response on a scale of 1-5 | The 4-mm straight needle was less painful and “scary” to inject vs. the 6-mm tapered needle |
| Nagai et al., 2013^103^ | Randomized, open-label crossover | 79 | Diabetes | - Straight: 32 G x 4 mm - Tapered: 33 G tip and 28 G base x 5 mm double-tapered wall | Pain on a 150 mm VAS | Patients reported less pain with the straight, 4-mm needle and reported that they preferred it to the longer needle (p<0.0001) |
| Narvarte & Rosset-Llobet, 2011^129^ | Prospective | 67 | Musculoskeletal complaints | 4- or 12-mm mesotherapy needle | Pain VAS and questionnaire about side effects | Participants reported pain with a larger proportion of injections but significantly less severity of pain with the 12-mm needle |
| Pager et al., 2020^133^ | Multicenter human factors formative study | 39 | At least one chronic disease, with or without experience administering self-injections and with or without hand impairment (rheumatoid arthritis or multiple sclerosis affecting fingers and/or hands) | 27 G, 8-mm staked needle with ultra-thin wall and 27 G 12.7-mm staked needle with special thin wall, both administered with prefillable glass syringe | VAS for anxiety, ease of pushing plunger rod, and acceptance of force required to push and patient report for preference and reason(s) for preference | 8-mm needle reported to have better ease of use and acceptance and to decrease injection-related anxiety compared with longer needle |
| Robb & Kanji, 2002^123^ | Prospective, randomized | 64 | Myocardial infarction (anticoagulant) | - 30 G x 8 mm - 26 G x 9.6 mm | - Hematoma diameter - Pain on a 10-point numeric scale (0=no pain, 10= worst possible pain) | No significant differences in pain or hematoma size |
| Ross et al., 1999^104^ | Prospective, open-label, nine-week, crossover study | 106 | Diabetes | - 8-mm 30 G pen needle - 12.7-m, 29 G pen needle | Pain on 10-point numeric scale (0= no pain, 10=extreme pain) | 8-mm needles were significantly less painful than 12.7-mm needle |
| Schwartz et al., 2004^105^ | Multi-center, open-label, randomized, two-period crossover study | 62 | Diabetes | - 6-mm 31 G - 12.7-mm 29 G | World Health Organization Diabetes Treatment Satisfaction Questionnaire (WHODTSQ), Insulin Treatment Satisfaction Questionnaire (ITSQ), Quality of Life Questionnaire (QOLQ), Needle Handling Questionnaire, 100-cm VAS for pain (0=no pain, 100=worst pain imaginable) | - Patients indicated greater satisfaction with shorter needle on the ITSQ (p<0.001) - No other significant differences in WHODTSQ or ITSQ scores - No significant difference in injection site bruising - 89% preferred the shorter needle (p<0.001) and 76% had a strong, very strong, or extremely strong preference for it - Slightly more patients rated longer needle as more likely to cause anxiety about using needles; significance not reported |
| Strauss et al., 1999^107^ | Prospective, controlled studies (six; four relevant for this review) | Pediatric study 1: 54  Pediatric study 2: 15  Adult study 1: 46  Adult study 2: 13 | Diabetes | 8-mm, 30 G  5-mm, 31 G  Pediatric study 1: pinch-up with 8-mm, no pinch-up with 5-mm  Pediatric study 2: pinch-up with both needles  Adult study 1: pinch-up with 8-mm, no pinch-up with 5-mm  Adult study 2: pinch-up with both needles | Not specified | - Pediatric study 1 - 52% preferred 5-mm needle, 35% preferred 8-mm needle, 13% had no preference - No significant differences in pain or site irritation - Pediatric study 2 - Significantly lower pain with 5-mm needle (p=0.001) - No differences in leakage or bleeding at the injection site - Preference for 5-mm needle over 8-mm needle (significance not specified) - Adult study 1 - 46% preferred 8-mm needle, 42% preferred 5-mm needle, 12% had no preference - No significant differences in pain, plunger pressure, leakage, or site irritation - Adult study 2 - No significant differences in pain, bleeding, bruising, or insulin leakage |
| van Dommelen et al., 2024^124^ | Retrospective, observational study | 862 | Growth disorders | - 29 G or 31 G - 4-, 6-, 8-, 10-, or 12-mm | Adherence | - % adherence in first 3 months: - 94% in patients with 6 mm injection depth, medium injection speed, 6-second injection time at treatment start - 95% in patients with 6 mm injection depth, slow injection speed, 6-second injection time at treatment start - 88% in patients with 4 mm injection depth, medium injection speed, and 6-second injection time at treatment start - Proportion of patients with high adherence was slightly lower in patients with a 4-mm injection depth, default injection speed (medium) and time (6 seconds) at treatment start compared with patients with all default settings (6 mm) or slow injection speed and default injection depth (6 mm) and time (p=0.02) and more often had to change settings during treatment (24% vs. 12%, p=0.002) |

Abbreviations: G, gauge; mm, millimeter; VAS, visual analogue scale.

**Supplementary Table 3: Summary of Articles Assessing the Impact of Needle Diameter on Pain, Anxiety, And Safety**

| **Reference** | **Study Design** | **N** | **Indication** | **Needle Characteristics** | **Outcome Measures** | **Key Findings** |
| --- | --- | --- | --- | --- | --- | --- |
| Arendt-Nielsen et al., 2006^73^ | Two blinded experiments | 30 in each study | Healthy volunteers | Experiment 1:   - 27 G - 28 G - 30 G - 31 G - 32 G   Experiment 2:   - 23 G - 25 G - 30 G | - ‘Pain’ vs. ‘no pain’ - Pain intensity on a 100 mm VAS - Bleeding incidence | - Positive correlation between needle diameter and frequency of painful injections (p<0.0001) - 31% of injections rated as painful with 32 G vs. 45% with 30 G, 50% with 28 G, 54% with 27 G - No impact on VAS score - Less bleeding with 31 G (10%) vs. 30 G (19%), 28 G (15%) or 27 G (25%) - Bleeding was associated with significantly higher pain VAS scores (p=0.004) |
| Boyd et al., 2008^122^ | Randomized, multicenter, open-label, parallel group | 60 | HIV | - 27 G - 31 G - NFID | - Patients (%) with Grade <2 ISR induration - Patients (%) with Grade <2 ISR:   - pain/discomfort   - erythema   - pruritis - bruising | No significant difference between groups for any ISR endpoint |
| Chan et al., 2004^128^ | Crossover | 54 | Thalassemia | - 27 G x 8 mm Thalaset needle - 25 G x 19 mm Butterfly scalp vein needle | - Pain, itchiness, tenderness and swelling assessed on a 5-point Likert scale | All four types of reaction were significantly more frequent with the 25 G needle vs. the 27 G needle |
| Coley et al., 1987^121^ | Single center, randomized, assessments were single-blinded | 73 | Postoperative thromboembolism (heparin) | - 25 G 5/8 inch needle (tuberculin) - 28 G ½ inch needle (insulin syringe) | - Hematoma presence/size - Pain Yes/no - Pain: none, mild, moderate, severe | - Hematoma occurred in 67.7% of patients in the tuberculin syringe group and 51.3% in the insulin syringe group (NS) - Pain occurred in 67.7% of patients in the tuberculin syringe group and 38.5% of patients in the insulin syringe group (26.2% vs. 15.2% of injections) - Pain severity was significantly lower in the insulin syringe group (13% of injections had mild pain vs. 22.5% with tuberculin; moderate pain was reported in 2.2% and 3.7% of injections respectively) |
| Egekvist et al., 1999^76^ | Randomized, partially blinded | 30 | Healthy volunteers | Insulin needles:   - 27 G - 30 G | - ‘Pain’ vs. ‘no pain’ - Pain intensity on a 100 mm VAS - Bleeding | - Significantly more bleeding with 27 G vs. 30 G (22.5% vs. 17.8%; p<0.05) - Significantly more pain with 27 G 40.3% vs. 30 G: 30.8%; p<0.05 - No impact of needle diameter on pain VAS - Visible bleeding was associated with pain |
| Gill et al., 2008^77^ | Randomized, double-blind | 10 | Healthy volunteers | - Microneedles with a thickness of 30, 45, and 100 µm and widths of 160, 245, and 465 µm - 26 G hypodermic needle | Pain intensity on a 100 mm VAS | - No difference in pain scores between microneedles of different diameters - Significantly less pain for microneedles with a thickness of 30, 45, and 100 µm and widths of 160, 245, and 465 µm versus a 26 gauge hypodermic needle inserted to 5 mm (pain reductions of 60–95%) |
| Glenski et al., 2009^111^ | Telephone surveys of patient preferences and experiences after switching to 29 G PFS | Survey 1: 659; survey 2: 564; survey 3: 562 | Multiple sclerosis | - PFS with a 29 G/5 bevel/0.5 inch needle with a TPE shield - 27 G | - Pain intensity on a 5-point scale of 0 (no pain) to 4 (severe pain) - Pain intensity compared with 27 G on a 5-point scale of +2 (a lot better) to -2 (a lot worse) - ISR on a scale of 0 (no ISR) to 4 (severe site reaction) - ISR compared with the 27 G on a scale of +2 (a lot better) to -2 (a lot worse) - Needle issues including bending or breaking | Most patients rated 29 G as less painful vs. 27 G:   - 27.5% rated it a lot better - 38.8% rated it a little better - 29.8% rated it as no different - 3.6% thought the 27 G was a little less painful - 0.4% thought 27 G was a lot less painful   Similarly, most patients thought the 29 G resulted in fewer ISR vs. 27 G:   - 16.7% rated it a lot better - 32.5% rated it a little better - 46.6% rated it as no different - 3.7% thought 27 G resulted in fewer ISR - 0.5% thought 27 G resulted in a lot fewer ISRs |
| Hanas et al., 2000^92^ | Double-blind, randomized | 60 (Study A)+ 40 (Study B) | Diabetes | - 27 G x 13 mm - 28 G x 13 mm - 28 G x 12 mm - 29 G x 13 mm - 30 G x 8 mm | - Pain on a 10 cm VAS - Leakage - Bleeding | - Bleeding was less frequent with 29 G 13 mm vs. 28 G x 12 mm needle (35.5 vs. 48.1%) - No statistically significant differences in pain scores between needles - Measured needle phobia at baseline but not in relation to needle characteristics |
| Iwanaga et al., 2009^96^ | Randomized, open-label, crossover | 30 | Diabetes | - 31 G, 0.25 mm tip, 5mm long straight - 32 G 0.23 mm tip, 6mm long tapered | 14-item questionnaire scored on a VAS of -100 (worst) to + 100 (best) covering:   - Pain - Brusing - Leakage | 32 G was significantly less frightening, less painful, yielded less frequent bleeding, bruising and leakage vs. the 31 G needle and had a higher overall satisfaction score |
| Jaber et al., 2008^80^ | Multiple studies including randomized, double-blind and surveys | 10 volunteers, 368 patients | Healthy volunteers and patients with multiple sclerosis | - 29 G /5-bevel needle with TPE shield - 27 G/3-bevel needle with TPE shield | Pain on 100 mm VAS | - 29 G/5-bevel needle with TPE shield was associated with 40% lower mean pain score than the 27 G/3-bevel needle with rubber shield (p < 0.01) - The mean VAS pain score was also 26% lower for the 29 G/5-bevel needle with the rubber shield than the 27 G/3-bevel needle with rubber shield (p = 0.0002) |
| McKay et al., 2009^100^ | Multicenter, randomized, open-label, two-period crossover trial | 119 | Diabetes | - 30 G 8 x mm - 32 G x 6 mm | - Pain yes/no - Pain intensity on a 0–100 mm VAS | - Pain perception was low, with 75% of all measurements scoring ≤12 mm on the 100 mm VAS - Pain-free injections more frequent with the 32 G needle vs. 30 G (24% vs 15%) - Little or no pain (VAS < 5 mm) more frequent with the 32 G needle vs. the 30 G (57% vs. 40%) |
| Miyakoshi et al., 2007^102^ | Single center, open-label, randomized, two-period crossover trial | 40 | Diabetes | - 31 G 5 mm non-tapered - 33 G 5 mm tapered | VAS of -100 (worst) to +100 (best) to assess:   - Fear - Pain - Ease of installing/removing the needle | 33 G was significantly less painful to insert, less frightening and caused less bruising vs. 31 G  VAS scores for 33 G vs. 31 G:   - Pain frequency during insertion 41.6 vs. 22.0 (p=0.030) - Frequency of bleeding 61.1 vs. 41.8 (p=0.072) - Frequency of bruising: 68.8 vs. 46.5 (p=0.025) |
| Nagai et al., 2013^103^ | Randomized, open-label crossover | 79 | Diabetes | 32 G x 4 mm Tapered: 33 G tip and 28 G base x 5 mm double-tapered wall | Pain on a 150 mm VAS | - 32 G needle was less painful (mean score  –14.5 mm; P<0.001) - 32 G needle was associated with significantly less bleeding 2.2% vs. 3.7% (P<0.0001) |
| Præstmark et al., 2016^34^ | Single-blind | 30 | Diabetes | 18 types of needles including: 32 G/3-bevel; 32 G tip 31 G base/3-bevel; 30 G/3-bevel; 28 G/3-bevel; 32 G/5-bevel; 34 G 34 G/3-bevel;  32 G/3-bevel;  32 G/2-bevel;  34 G/2-bevel;  32 G/1-bevel;  34 G/1-bevel;  32 G/1-bevel;  34 G/1-bevel | Pain scored on 100 mm VAS | Non-significant trend for decreased pain with smaller needles (least square mean pain scores ranged from 13.0 with a 32 G/5-bevel needle to 27.9 with a 32 G/2-bevel needle) |
| Robb and Kanji, 2002^123^ | Prospective, randomized | 64 | Myocardial infarction (anticoagulant) | - 30 G x 8 mm - 26 G x 9.6 mm | - Hematoma diameter - Pain on a 10-point numeric scale (0=no pain, 10= worst possible pain) | Reduced pain and hematoma formation with the 30 G needle (not significant)   - Mean pain score was 0.5 with the 26 G needle and 0.3 with the 30 G (P=0.10) - Pain was reported by 79% of patients in the 26 G group vs. 67% in the 30 G group - Hematoma formation in 70% of patients with the 30 G needle vs. 88% of patients with the 26 G needle |
| van Dommelen et al., 2024^124^ | Retrospective, observational study | 862 | Growth disorders | - 29 G - 31 G | Adherence | Adherence was similar in groups receiving injections with a 29 G needle or a 31 G needle |
| Wägo et al., 2016^78^ | Randomized | 36 | Healthy volunteers | - 21 G - 23 G - 27 G | Pain intensity on a 100 mm VAS | 27 G was reported as least painful by 58% of participants |

Abbreviations: ISR, injection site reaction; MS, multiple sclerosis; NFID, needle-free injection device; PFS, prefilled syringe; TPE, thermoplastic elastometer; VAS, visual analogue scale.

**Supplementary Table 4: Summary of Articles Assessing the Impact of Needle/Bevel Tip on Pain, Anxiety, and Safety**

| **Reference** | **Study Design** | **N** | **Indication** | **Needle Characteristics** | **Outcome Measures** | **Key Findings** |
| --- | --- | --- | --- | --- | --- | --- |
| Gill et al., 2008^77^ | Randomized, double-blind | 10 | Healthy volunteers | Microneedles:   - Tip angles of 20, 55 or 90 degrees and lengths of 480 or 960 µm | - Pain intensity on a 100 mm VAS | - No difference in pain scores between microneedles with different tip angles - Trend for lower pain with lower tip angles for the 480 µm but not 960 µm length needle |
| Hirsch et al., 2012a^33^ | Prospective, three-part, two-center | 86 | Diabetes | - 32 G x 4 mm 5-bevel - 31 G x 5 mm 5-bevel - 31 G x 8 mm 5-bevel - 32 G x 6 mm 3-bevel - 31 G x 6 mm 3-bevel - 30 G x 8 mm 3-bevel | - Acceptability - Pain on a 15 cm VAS | - No difference in pain scores between different tip designs during blinded phase - Significantly lower mean pain VAS for 5-bevel vs. 3-bevel tips during at home use |
| Jaber et al., 2008^80^ | Single-center | 10 | Healthy volunteers | - 29 G /5-bevel needle with TPE shield - 29 G /3-bevel needle with TPE shield - 29 G /5-bevel needle with rubber shield - 29 G /3-bevel needle with rubber shield - 29 G /5-bevel needle with TPE shield - 27 G /3-bevel needle with TPE shield - 27 G /5-bevel needle with rubber shield - 27 G /3-bevel needle with rubber shield | - Pain on 100 mm VAS | The mean VAS pain score was slightly but consistently lower for 5 vs. 3 bevel design |
| Omoigui et al., 2006^125^ | Prospective | 20 | Unknown – anesthetic | - BD Precisionglide needles: 25 G, 15.9 mm long, low-angle bevel - Terumo needle: 25 G, 15.9 mm long, double bevel | Pain on a 10-point scale (0=least pain; 10=worst pain) | - Less pain with the BD Precisionglide needles vs. Terumo needles (mean pain score 1.9 vs. 5.5) - Minimum and maximum pain scores were 0 and 6 with the BD needle vs. 3 and 10 with the Terumo |
| Præstmark et al., 2016^34^ | Single-blind | 30 | Diabetes | 18 types of needles including: 32 G/3-bevel; 32 G tip 31 G base/3-bevel; 30 G/3-bevel; 28 G/3-bevel; 32 G/5-bevel; 34 G 34 G/3-bevel;  32 G/3-bevel;  32 G/2-bevel;  34 G/2-bevel;  32 G/1-bevel;  34 G/1-bevel;  32 G/1-bevel;  34 G/1-bevel | Pain scored on 100 mm VAS | Higher pain intensity for the 2-bevel 32 G needle versus the 32 G 3-bevel needle tip |

Abbreviations: G, gauge; mm, millimeters; TPE, thermo plastic elastomer; VAS, visual analogue scale

**Supplementary Table 5: Summary of Articles Assessing the Impact of Needle Tapering on Pain, Anxiety, And Safety**

| **Reference** | **Study Design** | **N** | **Indication** | **Needle Characteristics** | **Outcome Measures** | **Key Findings** |
| --- | --- | --- | --- | --- | --- | --- |
| Asakura et al., 2006^89^ | Randomized, crossover | 99 | Diabetes | - Tapered from 33 G to 28 G - 31 G, 5 mm long, thin-walled | Pain on a 10-cm VAS | - 44.4% of patients preferred a microtapered needle; 18.8% preferred the comparison needle, 36.7% found no difference between the two needles - The tapered needle resulted in significantly less pain (mean VAS 1.48 vs. 2.47; P<0.001) |
| Iwanaga et al., 2009^96^ | Randomized, open-label, crossover | 30 | Diabetes | - 31 G, 0.25 mm tip, 5mm long straight - Tapered: 32 G 0.23 mm tip, 6mm long | 14-item questionnaire scored on a VAS of -100 (worst) to + 100 (best) covering:   - Pain - Brusing - Leakage | 32 G was significantly less frightening, less painful, yielded less frequent bleeding, bruising and leakage vs. the 31 G needle and had a higher overall satisfaction score |
| Miyakoshi et al., 2007^102^ | Single center, open-label, randomized, two-period crossover trial | 40 | Diabetes | - 31 G 5 mm non-tapered - 33 G 5 mm tapered | VAS of -100 (worst) to +100 (best) to assess:   - Fear - Pain - Ease of installing/removing the needle | Tapered was significantly less painful to insert, less frightening and caused less bruising vs. non-tapered  VAS scores for tapered vs. non-tapered:   - Pain frequency during insertion 41.6 vs. 22.0 (p=0.030) - Frequency of bleeding 61.1 vs. 41.8 (p=0.072)   Frequency of bruising: 68.8 vs. 46.5 (p=0.025) |
| Nagai et al., 2013^103^ | Randomized, open-label crossover | 79 | Diabetes | - 32 G x 4 mm - Tapered: 33 G tip and 28 G base x 5 mm double-tapered wall | Pain on a 150 mm VAS | - 32 G needle was less painful (mean score  –14.5 mm; P<0.001) - 32 G needle was associated with significantly less bleeding 2.2% vs. 3.7% (P<0.0001) |

Abbreviations: G, gauge; mm, millimeters; VAS, visual analogue scale.

**Supplementary Table 6: Summary of Articles Assessing the Impact of Needle Wall Thickness on Pain, Anxiety, and Safety**

| **Reference** | **Study Design** | **N** | **Indication** | **Needle Characteristics** | **Outcome Measures** | **Key Findings** |
| --- | --- | --- | --- | --- | --- | --- |
| Aronson et al., 2013^88^ | Prospective, randomized, open-label, crossover trial | 198 | Diabetes | - Pen needle with an extra-thin wall (32 G x 4 mm, 31 G x 5 mm, and 31 G x 8 mm - Three standard pen needles 32 G x 4 to 8 mm; 31 G x 4.5 to 6 mm; and 31 G x 8 mm | Thumb force, confidence in delivering the full dose, perceived time to deliver insulin on a 150-mm VAS | Pen needles with an extra-thin wall were preferred overall and on each individual score including thumb force for injection, confidence that the entire dose was delivered, injection time, pain, insertion ease and convenience of use |
| Gill et al., 2008^77^ | Randomized, double-blind | 10 | Healthy volunteers | Microneedles with thicknesses of 30, 45, and 100 μm | Pain intensity on a 100-mm VAS | No significant effect on pain scores of increasing microneedle thickness by >3-fold (p=0.6) |
| Siegmund et al., 2009^106^ | Multicenter, open-label, single-arm | 97 | Diabetes | - 31 G “thin-wall” pen needle - 31 G “regular-wall” pen needle | Standardized questionnaire assessing injection pain, skin irritation, bleeding, leakage, needle blockage, and patient satisfaction | Significantly higher proportion of patients reporting less pain, bleeding, skin irritation, injection strain, residual insulin leakage after injection, and needle occlusion with use of a “thin-wall” needle |

Abbreviations: G, gauge; μm, micrometers; mm, millimeters; VAS, visual analogue scale.

**Supplementary Table 7: Summary of Articles Assessing the Impact of Microneedles on Pain, Anxiety, And Safety**

| **Reference** | **Study Design** | **N** | **Indication** | **Needle Characteristics** | **Outcome Measures** | **Key Findings** |
| --- | --- | --- | --- | --- | --- | --- |
| Gill et al., 2008^77^ | Randomized, double-blind | 10 | Healthy volunteers | - Microneedles with a thickness of 30, 45, and 100 µm and widths of 160, 245, and 465 µm - 26 G hypodermic needle | Pain intensity on a 100 mm VAS | - No difference in pain scores between microneedles of different diameters - Significantly less pain for microneedles with a thickness of 30, 45, and 100 µm and widths of 160, 245, and 465 µm versus a 26 gauge hypodermic needle inserted to 5 mm (pain reductions of 60–95%) |
| Lee et al., 2018^132^ | Not specified | 40 | Volunteers, of whom 15 had type 2 diabetes for evaluation of pharmacodynamic profile of insulin (data not shown) | - 4-mm long, 120 µm in outer diameter microneedle with 15° sharp bevel angle - 32 G, 4-mm pen needle with 230-µm outer diameter | Numerical Rating Scale (0=no pain, 2=mild pain, 4=moderate pain) | - Significantly less pain with microneedle (no to mild pain) compared with pen needle (mild pain) - Less bleeding at injection site with microneedle (0 events) compared with pen needle (3 events) |

Abbreviations: G, gauge; μm, micrometers; mm, millimeters; VAS, visual analogue scale.

**Supplementary Table 8: Summary of Articles Assessing the Impact of SC Infusion Needle Characteristics on Pain, Anxiety, And Safety**

| **Reference** | **Study Design** | **N** | **Indication** | **Needle Characteristics** | **Outcome Measures** | **Key Findings** |
| --- | --- | --- | --- | --- | --- | --- |
| Abbas et al., 2005^81^ | Observational | 40 | Palliative care | - Metal needle with battery-driven portable syringe driver - Plastic needle (22 or 26 G) with battery-driven portable syringe driver | Injection site reactions | Greater number of complications with metal needles (37.5% vs. 19.6% with plastic, all inflammation) |
| Chan et al., 2004^128^ | Crossover | 54 | Thalassemia | - 27 G x 8 mm Thalaset needle - 25 G x 19 mm butterfly scalp vein needle | Pain, itchiness, tenderness and swelling assessed on a 5-point Likert scale | All four types of reaction were significantly more frequent with the butterfly needle vs. the Thalaset needle |
| Currow and Cooney, 1994^82^ | Randomized trial | 63 | Palliative care | - Metal butterfly needle (25 G) - Winged plastic 24 G cannula | Injection site reactions | Metal needles were significantly associated with more local reactions (9/30 vs. 2/33; p=0.04) |
| Dawkins et al., 2000^127^ | Randomized, parallel | 42 | Oncology | - Metal butterfly needle - Winged plastic cannula | Injection site reactions including redness, swelling and tenderness (rated 0=none to 3=severe)  Cannula problems (yes/no) including skin puncture, needlestick injury, blocked or kinked cannula, accidental removal and leakage | - Plastic cannulas were associated with fewer injection site reactions vs. metal needles - 23/50 metal needle insertions were re-sited due to reactions vs. 13/50 insertions with the plastic needle |
| Macmillan et al., 1994^83^ | Prospective, randomized, crossover | 20 | Palliative care | - Metal butterfly needle (25 G) - Winged Teflon cannula (26 G and 24 G cannula) | Injection site reactions, comfort | Teflon cannula was preferred by patients due to decreased need for injection site changes but there was no difference in comfort between the two |
| Neo et al., 2016^84^ | Observational | 27 | Palliative care | - Winged chrome-nickel steel 23 G needle , - Winged 24 G cannula - Nonwinged polyurethane cannula | Pain (numeric pain rating scale 0 to 10), bruising, blister and erythema swelling and mechanical complications | The metal needle resulted in pain in 1/10 patients (score of 3), blister and erythema occurred in 1/10 patients and swelling in 3/10 vs. no patients with the other cannula types |
| Youssef and Atkinson, 1990^85^ | Randomized | 20 | Palliative care | - Metal butterfly needle - Winged Teflon cannula | Injection site reactions | Significantly lower incidence of swelling with the Teflon cannula (p<0.05) |

Abbreviations: G, gauge; mm, millimeters.

**Supplementary Table 9: Summary of Articles Assessing the Impact of Device Choice on Pain, Anxiety, And Safety**

| **Reference** | **Study Design** | **N** | **Indication** | **Needle Characteristics** | **Outcome Measures** | **Key Findings** |
| --- | --- | --- | --- | --- | --- | --- |
| Alten et al., 2020^116^ | Systematic pooled analysis of four studies | 1001 | Rheumatoid arthritis | - Autoinjector - PFS | - Pain on a 100 mm VAS - Injection site reactions (classed as tenderness, erythema, induration) which were measured along the longest axis and graded 0-7 where 0 is no evidence of irritation and 7 is strong reaction spreading beyond test site) | - VAS pain score with an autoinjector was 4.4 points lower vs. a vial/syringe (p<0.001), and 1.7 points lower vs. a PFS (p=0.035) - VAS pain scores were 2.6 points lower with a PFS than with a vial/syringe (p=0.002) |
| Borrás-Blasco et al., 2010^114^ | Two-phase, cross-sectional study | 51 in the second phase of the study | Rheumatic diseases | - Autoinjector - PFS | VAS pain on a 10-point scale where 0=no pain and 10 was the worst pain imaginable | - Injection site pain was significantly reduced with the autoinjector - Mean VAS was 3.52 for the PFS vs. 2.02 for the autoinjector (p<0.001) |
| Kadiri et al., 1998^97^ | Randomized, open-label, multicenter cross-over | 78 | Diabetes | - Autoinjector - Vial/syringe | Questionnaire relating to dosing, injection pain and acceptance | Autoinjector was clinically and significantly less painful than the vial/syringe (p=0.0018) |
| Kivitz et al., 2006^117^ | Multicenter, open-label, single-arm sequential | 52 | Rheumatoid arthritis | - Autoinjector - PFS | Pain on an 11-point scale (0= no pain) to 10 (pain as bad as it could be) | - 76.9% found the autoinjector less painful vs. the PFS - 7.7% found the PFS less painful - 15.4% had no preference |
| Lim et al., 2012^126^ | Randomized, crossover | 50 | Chronic kidney disease | - Autoinjector - PFS | VAS and Likert scale questionnaires to assess type, severity and duration of pain | - No difference in pain scores when switching from a PFS to an autoinjector - 62% of patients preferred the autoinjector - No significant difference in anxiety or pain between autoinjector and PFS |
| Ramael et al., 2018^134^ | Randomized, open-label, parallel-group | 233 | Healthy volunteers | - Autoinjector - PFS | Injection site reactions | 32.1% of patients in the autoinjector group reported injection site reactions vs. 24.7% with the PFS |
| Saraux et al., 2019^119^ | Randomized, open-label, parallel group | 202 | Rheumatoid arthritis | - Autoinjector - PFS | - VAS pain - Adherence (actual injections/planned injections) - Health assessment questionnaire - Disease activity - QoL | - 72.6% of patients rated the autoinjector as not at all or somewhat not painful vs. 69.2% with the PFS - Autoinjector was rated as not at all stressful or somewhat not stressful by 77.4% of patients vs. 66.6% with the PFS - No significant difference observed regarding injection-related pain or stress |
| Zhang et al., 2021^79^ | Randomized, open-label | 180 | Healthy volunteers | - Autoinjector - PFS | Injection site reactions | - Injection site reactions were reported in 33.3% of volunteers with the autoinjector vs. 26.7% with the PFS |

Abbreviations: G, gauge; mm, millimeters; PFS, pre-filled syringe; QoL, quality of life; VAS, visual analogue scale.

**Supplementary Table 10**: **Summary of Articles Assessing the Impact of a Hidden/Retractable Needle on Pain, Anxiety, and Safety**

| **Reference** | **Study Design** | **N** | **Indication** | **Needle Characteristics** | **Outcome Measures** | **Key Findings** |
| --- | --- | --- | --- | --- | --- | --- |
| Al Hayek et al., 2021a^86^ | Prospective | 72 | Diabetes | - Safety pen containing a 31 G, 6 mm long, 250 µm diameter needle - 31 G, 6 mm long, 250 µm diameter needle | - 14-question usability scale covering 6 domains (pain /fear based on needle appearance, installing and removing the needle, pain and sticking from inserting the needle, bleeding and bruising, dribbling of injected insulin, and power of pushing button, overall satisfaction) each scored on a 10-item VAS - Diabetes Fear of Injecting and Self-Testing Questionnaire (D-FISQ) scored on a 4-point Likert scale where 1=never / almost never and 4=almost always or always | - Safety pen significantly reduced fear and frequency and intensity of pain, injection site bleeding and bruising (all p<0.01) - Significant decrease in all aspects of fear of self-injection including being restless, afraid, worried and nervous |
| D’Arcy et al., 2012^110^ | Phase IV, observational, open-label, single-arm | 59 | Multiple sclerosis | Electric autoinjector (29 G, 12 mm long, 330 µm diameter hidden needle) | - 10 cm VAS to rate how much they liked the device - 4-point response question on ease of device use - List of 10 device functions to rank (1-10) based on most/least useful | - 91.5% (n=54/59) of patients reported that they liked using the autoinjector (VAS score of ≥6) - Hidden needle was ranked 1^st^ as the most useful device feature |
| Devonshire et al., 2016^113^ | Phase IV observational, single-arm | 162 | Relapsing multiple sclerosis | Electric autoinjector (29 G, 12 mm long, 330 µm diameter hidden needle) | - Anxiety on the HADS (14-item anxiety and depression scale) - Anxiety on the STAI (temporary, situational anxiety and daily, non-situational anxiety) - 16-item patient experience questionnaire | - 49.6% reported no pain / mild pain - 69.9% were less fearful of injections after 6 months vs. study start - Significant reduction from baseline in proportion of patients with injection fear (48.3% vs. 16.0%) - Significant reduction from baseline in pre-injection state anxiety using both assessments |
| Lamblet et al., 2011^131^ | Randomized, multicenter clinical trial | 240 | Indication not specified; insulin injected | - 26 G, 13 mm long, 450 µm diameter conventional needle (n=120) - Retractable 27 G 12.7 mm long, 400 µm diameter needle | - Pain intensity on a scale of 0 to 10 in the pooled intramuscular and subcutaneous injection groups - Bruising | - Conventional needle resulted in significantly greater bruising 24 hours following administration (p=0.029) |
| Pivato et al., 2024^130^ | Cross-sectional observational study | 111 | Chronic rheumatic, gastrointestinal, or dermatologic disease being treated with self-administered biologic available as prefilled pen or prefilled syringe | - Prefilled syringe - Prefilled pen | Web-based questionnaire:   - Reason for preference from a list: habit, ease of use, reduced pain sensation, better administration and needle-free vision | - 46 patients preferred pre-filled syringe - 65 preferred pre-filled pens - Hidden needle was the reason for choosing the pre-filled pen in 23.1% of participants |

Abbreviations: G, gauge; HAD, Hospital Anxiety and Depression Scale; µm, micrometers; mm, millimeters; STAI, state-trait anxiety inventory; VAS, visual analogue scale.

**Supplementary Table 11: Summary of Articles Assessing the Impact of Injection Administration on Pain, Anxiety, And Safety**

| **Reference** | **Study Design** | **N** | **Indication** | **Needle Characteristics** | **Outcome Measures** | **Key Findings** |
| --- | --- | --- | --- | --- | --- | --- |
| Candiotti et al., 2009^74^ | Prospective, randomized | 50 | Healthy volunteers | 29 G needle | Verbal rating score to quantify pain sensation on a scale of 0 to 10 (where 0=no pain and 10= worst pain) | Needle bevel positioned up was associated with significantly decreased pain vs. bevel positioned down |
| Egekvist et al., 1999^76^ | Randomized, partially blinded | 30 | Healthy volunteers | - 8 mm - 27 G or 30 G - 2 or 19 mm/second - 45° or 90° angle of insertion | - ‘Pain’ vs. ‘no pain’ - Pain intensity on a 100 mm VAS - Bleeding | - No significant difference in occurrence of pain between 2 and 19 mm/second insertion velocity or 45° or 90° angle of insertion - 19 mm/second insertion velocity associated with higher rate of sharp and pricking pain - 2 mm/second insertion velocity associated with higher rate of dull and burning pain |
| Müller-Ladner et al., 2010^118^ | Open-label, comparative, within-patient controlled, multicenter study | 132 | Rheumatoid arthritis | Two pen needles, one with a separate needle and one with a pre-attached needle | Questionnaires and VASes | 89.1% assessed the usability of having a  pre-attached needle with the small syringe (HC formulation)  as an “advantage” and “great advantage” and 3.1% as a  “disadvantage” and “great disadvantage” |
| Striesow and Brandt, 2012^120^ | Cross-sectional post-marketing surveillance study | 403 | Rheumatoid or psoriatic arthritis | - SC high-concentration ready-to-use methotrexate prefilled syringe with pre-attached needle | Questionnaires | - 91.8% of patients considered the availability and use of the pre-attached needle as very advantageous or advantageous - 96% described the injection as comfortable or tolerable - 89.1% reported that self-administration led to feeling of more independence and 83.6% said it led to improved quality of life |

Abbreviations: G, gauge; IM, intramuscular; mm, millimeters; SC, subcutaneous; VAS, visual analogue scale.

**Supplementary Table 12. Risk of Bias Appraisal for Cross-Sectional Studies with the Joanna Briggs Institute (JBI) Checklist for Analytical Cross-Sectional Studies**

| **Question** | **Reference** | | | | |
| --- | --- | --- | --- | --- | --- |
|  | **Arendt-Nielsen et al., 2006^73^** | **Borrás-Blasco et al., 2010^114^** | **D’Arcy et al., 2012^110^** | **Pivato et al., 2024^130^** | **Striesow and Brandt, 2012^120^** |
| 1. Were the criteria for inclusion in the sample clearly defined? | No | Yes | Yes | Yes | Yes |
| 2. Were the study subjects and the setting described in detail? | No | Yes | No | Yes | Yes |
| 3. Was the exposure measured in a valid and reliable way? | Yes | Yes | Yes | Yes | Yes |
| 4. Were objective, standard criteria used for measurement of the condition? | Yes | Yes | Yes | Yes | Yes |
| 5. Were confounding factors identified? | Yes | No | No | No | No |
| 6. Were strategies to deal with confounding factors stated? | Yes | No | No | No | No |
| 7. Were the outcomes measured in a valid and reliable way? | Yes | Yes | Yes | Yes | Yes |
| 8. Was appropriate statistical analysis used? | Yes | Yes | Yes | Yes | No |
| **Overall Appraisal** | **Include** | **Include** | **Include** | **Include** | **Include** |

**Supplementary Table 13. Risk of Bias Assessment for Cohort Studies Using the Newcastle-Ottawa Quality Assessment Scale (NOS) – Cohort Studies**

| **Reference** | **van Dommelen et al.^124^** | | |
| --- | --- | --- | --- |
| **Domain** | **Item** | **Score** | **Total/Possible** |
| **Selection** | Representativeness of the exposed cohort | - | **/**** |
|  | Selection of the non-exposed cohort | - |  |
|  | Ascertainment of exposure | * |  |
|  | Demonstration that outcome of interest was not present at start | * |  |
| **Comparability** | Comparability of cohorts on the basis of design or analysis | * | */** |
| **Outcome/Exposure** | Assessment of outcome | * | ***/*** |
|  | Was follow-up long enough for outcomes to occur? | * |  |
|  | Adequacy of follow-up of cohorts | * |  |
| **Total** |  |  | 7/9 (Good Quality) |

**Supplementary Table 14. Risk of Bias Appraisal for Case Series with JBI Critical Appraisal Checklist for Case Series**

|  | **Reference** |
| --- | --- |
| **Question** | **Abbas et al., 2005^81^** |
|  | **Appraisal** |
| Were there clear criteria for inclusion in the case series? | No |
| Was the condition measured in a standard, reliable way for all participants included in the case series? | Yes |
| Were valid methods used for identification of the condition for all participants included in the case series? | Not applicable |
| Did the case series have consecutive inclusion of participants? | Unclear |
| Did the case series have complete inclusion of participants? | Unclear |
| Was there clear reporting of the demographics of participants in the study? | Yes |
| Was there clear reporting of clinical information of the participants? | No |
| Were the outcomes or follow up results of cases clearly reported? | Yes |
| Was there clear reporting of the presenting site(s)/clinic(s) demographic information? | No |
| Was statistical analysis appropriate? | Yes |
| **Overall Appraisal** | **Include** |

**Supplementary Table 15. Qualitative Assessment of Certainty of Evidence by Outcome**

| **Outcome** | **Studies Contributing Evidence** | **Overall Direction of Findings** | **Major Limitations** | **Confidence in Evidence** |
| --- | --- | --- | --- | --- |
| Pain | Most frequently assessed outcome; evaluated across needle diameter, length, tapering, bevel/tip design, wall thickness, microneedles, subcutaneous infusion needles, device choice, and administration factors | Generally favored smaller diameter, shorter length, microneedles, plastic/Teflon cannulas over metal needles and autoinjectors over prefilled syringes/vial-syringe systems in several studies, although not all studies reported significant differences | Most studies were open-label; pain was usually patient-reported; frequent confounding between needle characteristics (e.g., length, diameter, tapering, wall thickness); all randomized studies had at least some concerns for risk of bias | Higher confidence |
| Anxiety/fear/stress/phobia | Assessed in a relatively small number of studies, primarily involving concealed/retractable needles, device characteristics, and selected needle designs | Available evidence favored concealed needles and some shorter, thinner, or tapered needle designs | Fewer studies; validated anxiety instruments used inconsistently; many outcomes based on study-specific fear or preference measures; most contributing non-randomized studies were judged to have risk of bias | Less confidence |
| Injection site safety outcomes (e.g., bleeding, bruising, leakage, injection site reactions) | Frequently assessed across needle length, diameter, subcutaneous infusion needles, concealed needles, and device studies | Many studies reported improvements with shorter or thinner needles and plastic/Teflon cannulas, but findings varied by specific outcome | Substantial heterogeneity in outcome definitions and assessment methods; many studies compared interventions differing in multiple characteristics simultaneously; objective and subjective outcomes often combined | Less confidence |
| Usability, satisfaction, and patient preference | Commonly assessed across needle, device, and administration studies | Generally favored shorter, thinner, concealed/retractable, thin-wall, autoinjector, and pre-attached needle systems | Outcomes were predominantly subjective, frequently assessed using non-validated instruments, and highly susceptible to expectation, novelty, and performance bias in unblinded studies | Less confidence |
| Adherence | Infrequently assessed | Some studies reported improved adherence with shorter and thinner needles or selected device features | Small evidence base; observational evidence common; adherence definitions varied; potential confounding from patient, disease, and device-related factors | Less confidence |

**Supplementary Figure I. ROBINS-I risk-of-bias traffic light plot**

**Supplementary Figure 2. ROBINS-I risk-of-bias summary plot**

**Supplementary Figure 3. RoB 2 risk-of-bias traffic light plot**

**Supplementary Figure 4. RoB 2 risk-of-bias summary plot**
